# Modulation of the Gut Microbiota by Prebiotics, Probiotics, and Psychobiotics and Its Impact on the Gut Microbiota–Brain Axis: A Systematic Review

**DOI:** 10.3390/biology15181598

**Published:** 2026-09-10

**Authors:** Santiago Revelo, Miguel Anchundia

**Affiliations:** 1 Applied Microbiology Program, Universidad Politécnica Estatal del Carchi, Tulcán 040102, Carchi, Ecuador; 2 Food Engineering Program, Universidad Politécnica Estatal del Carchi, Tulcán 040102, Carchi, Ecuador; miguel.anchundia@upec.edu.ec

**Keywords:** gut microbiota–brain axis, psychobiotics, probiotics, prebiotics, neuroinflammation

## Abstract

Background: The gut microbiota–brain axis is a highly integrated bidirectional communication network operating through neural, neuroendocrine, immune, and metabolic pathways that maintain central nervous system homeostasis and has emerged as a promising complementary therapeutic target for neurological and neuropsychiatric disorders. Objective: The objective of this study is to systematically evaluate the effects of prebiotics, probiotics, and psychobiotics on gut–brain communication and neurological and behavioral outcomes, including neuroinflammatory markers, HPA-axis parameters, and microbial metabolites. Methods: A systematic review was conducted in accordance with the PRISMA 2020 guidelines. Literature searches of PubMed, Scopus, and Google Scholar from 2020 up to March 2026 identified 81 eligible studies (48 preclinical studies, 27 randomized controlled trials, and 6 quasi-experimental clinical studies) from 7358 records. Primary outcomes were systematically categorized into four domains: (1) cognitive performance and social/adaptive behavior; (2) neuroinflammatory markers (TNF-α, IL-6, IL-1β) and barrier integrity; (3) HPA-axis parameters (cortisol/corticosterone); and (4) neuroactive microbial metabolites (short-chain fatty acids and tryptophan derivatives). Methodological quality and risk of bias were assessed independently by two reviewers using the SYRCLE tool for preclinical studies and the Joanna Briggs Institute (JBI) tools for randomized and quasi-experimental clinical trials, with summary visualizations generated using the robvis web application (version 0.3.0). Results: Neurodegenerative diseases (*n* = 31; 38.3%) and mood disorders (*n* = 29; 35.8%) were the most frequently investigated conditions. Probiotics predominated (*n* = 61), followed by prebiotics (*n* = 12) and synbiotics (*n* = 8). Descriptively, 92% of included studies reported improvements in at least one evaluated outcome. However, this unweighted observation reflects effect direction rather than clinical magnitude, encompassing primary and secondary endpoints across highly heterogeneous sample sizes, study designs, and risk-of-bias profiles. Conclusions: Microbiota-targeted interventions show promise as complementary strategies for neurological disorders; however, substantial methodological heterogeneity, unstandardized dosing, and the absence of quantitative meta-analysis preclude definitive clinical recommendations. Successful translation will require harmonized protocols, strain-specific functional characterization, and precision microbiota-based trials.

## Simple Summary

The gut microbiota–brain axis is a highly integrated bidirectional communication network that plays a fundamental role in maintaining central nervous system homeostasis and regulating neurological function, cognition, and behavior. However, uncertainty remains regarding the effectiveness of microbiota-targeted interventions for improving neurological outcomes through modulation of the gut microbiota. This systematic review analyzed 81 original studies (48 preclinical studies, 27 randomized clinical trials, and six non-randomized clinical intervention studies) to evaluate the effects of prebiotics, probiotics, and psychobiotics on the gut microbiota and gut–brain communication. The available evidence suggests that these interventions may alleviate symptoms of neurodegenerative diseases and mood disorders by modulating neuroendocrine, immune, and metabolic pathways implicated in neuroinflammation. Overall, this review provides a comprehensive synthesis of the therapeutic potential of prebiotics, probiotics, and psychobiotics as complementary interventions, highlighting the emerging role of psychobiotics as strain-specific therapies and the need to move beyond empirical dosing strategies toward precision microbiota-based interventions supported by high-quality clinical evidence.

## 1. Introduction

The gut microbiota comprises the community of microorganisms that inhabit the gastrointestinal tract and establish a symbiotic relationship with the host [1]. Although the microbial-to-human cell ratio was historically estimated at approximately 10:1, more recent evidence suggests that it is closer to 1:1. This complex microbial ecosystem is predominantly composed of four phyla—*Proteobacteria*, *Firmicutes*, *Actinobacteriota*, and *Bacteroidota*—which account for 93.5% of the human-isolated microbial species described to date [2]. The establishment of this ecosystem is a dynamic process that begins at birth through maternal microbial transmission, during which breast milk selectively promotes the predominance of *Bifidobacterium* spp. [3]. Thereafter, the composition of the gut microbiota continues to evolve throughout life under the influence of multiple factors, including age, diet, lifestyle, and antibiotic exposure [1].

Functionally, the gut microbiota behaves as a metabolically active organ that contributes to host homeostasis through metabolic, immunological, and barrier-protective functions [4]. Metabolically, it ferments nondigestible carbohydrates into short-chain fatty acids (SCFAs), including acetate, propionate, and butyrate, which play essential roles in energy metabolism and the regulation of glucose and lipid homeostasis. In addition, the gut microbiota contributes to vitamin biosynthesis, mineral absorption, and the production of neuroactive compounds, including serotonin and γ-aminobutyric acid (GABA). Since peripheral serotonin cannot cross the blood–brain barrier, microbial modulation of central nervous function relies on indirect vagal, enteric, and metabolic signaling [5]. It also preserves intestinal barrier integrity and promotes immune system maturation, thereby limiting excessive inflammatory responses and maintaining intestinal homeostasis [5].

Disruption of this microbial equilibrium, commonly referred to as dysbiosis, has been associated with a wide range of intestinal and extraintestinal disorders and may contribute to—or result from—immunological, metabolic, and neuropsychiatric diseases [6,7]. Dysbiosis compromises intestinal barrier integrity, increasing intestinal permeability and facilitating the translocation of bacterial endotoxins, particularly lipopolysaccharides (LPS), along with other pathogen-associated molecular patterns (PAMPs), into the systemic circulation. The subsequent activation of innate immune signaling promotes the release of pro-inflammatory mediators, ultimately contributing to chronic low-grade systemic inflammation, a hallmark of numerous chronic disorders [8].

The systemic consequences of chronic low-grade inflammation extend far beyond the gastrointestinal tract. Metabolically, persistent inflammation disrupts energy homeostasis and contributes to the development of insulin resistance, obesity, and type 2 diabetes mellitus [9]. Immunologically, it impairs immune regulation, thereby increasing susceptibility to allergic and autoimmune diseases. In addition, circulating inflammatory mediators can cross or alter the integrity of the blood–brain barrier, promoting neuroinflammation, disrupting neurotransmitter homeostasis, and increasing the risk of neuropsychiatric disorders and behavioral dysfunction [10].

Bidirectional communication between the gut microbiota and the central nervous system (CNS), collectively known as the gut microbiota–brain axis, constitutes a complex signaling network that regulates neurological, psychological, and behavioral functions [1]. Experimental studies in animal models have demonstrated that the absence of gut microorganisms disrupts neurodevelopment, alters stress responsiveness, and impairs neuronal plasticity. Notably, many of these alterations can be partially reversed through early-life microbial colonization or fecal microbiota transplantation [10,11]. In humans, growing evidence indicates that probiotics and psychobiotics can modulate mood, stress responsiveness, and cognitive function [12]. These observations highlight the functional relevance of the gut microbiota–brain axis, whose bidirectional signaling is mediated primarily by four interconnected biological pathways.

The neuronal pathway constitutes the fastest interaction route and is mediated primarily by the enteric nervous system and the vagus nerve [11]. In parallel, the endocrine pathway involves activation of the hypothalamic–pituitary–adrenal (HPA) axis together with the secretion of hormones and neuroactive peptides by enteroendocrine cells, thereby establishing a key regulatory interface between the gastrointestinal tract and the central nervous system [1,12].

The immune pathway is driven by the gut microbiota’s ability to modulate peripheral immune responses by regulating cytokine production and immune cell activation. These immune mediators indirectly communicate with the central nervous system, shaping neuroinflammatory processes and influencing brain function [8]. The metabolic pathway links the gut and the brain through the production of microbially derived metabolites, including short-chain fatty acids (SCFAs), tryptophan metabolites, and other neuroactive compounds. These metabolites influence neurological function either by directly interacting with the central nervous system or by activating peripheral signaling pathways involved in gut–brain communication [11,12]. Notably, these signaling pathways are context-dependent, being modulated by host factors such as age, baseline clinical status, and donor-related variability in microbiota-based interventions.

Given the dynamic nature of the gut microbiota, several strategies have been explored to restore eubiosis through prebiotics, probiotics, and targeted synbiotic formulations [9,13,14]. Although psychobiotics were originally defined as specialized live organisms conferring mental health benefits, the concept has evolved to encompass any microbiota-targeted intervention including specific bacterial strains and prebiotic substrates capable of modulating gut microbiota–brain axis signaling when administered at adequate doses [1,11].

Despite the growing body of research on gut microbiota modulation through prebiotics, probiotics, and psychobiotics, the available evidence remains heterogeneous and fragmented due to differences between preclinical and clinical studies, experimental designs, intervention strategies, microbial strains, and outcome measures. A fundamental translational gap persists between preclinical animal models which primarily demonstrate biological plausibility and neurochemical pathways and clinical human trials evaluating functional outcomes.

In this context, a systematic review is needed to critically synthesize the available evidence on the effects of prebiotics, probiotics, and psychobiotics on gut microbiota–brain axis communication. Such an approach provides a comprehensive framework for guiding future research and supporting the development of evidence-based therapeutic strategies.

## 2. Materials and Methods

### 2.1. Study Design and Protocol Registration

This systematic review was conducted in accordance with the Preferred Reporting Items for Systematic Reviews and Meta-Analyses (PRISMA 2020) guidelines [15] and synthesized the available evidence using a narrative approach. The review protocol was prospectively registered in the Open Science Framework (OSF) and is publicly available at https://doi.org/10.17605/OSF.IO/SBVD9 (accessed on 7 March 2026).

The core research question was formulated using the PECO (Population, Exposure, Comparison, and Outcome) framework as follows:

Population (P): Humans and in vivo animal models with characterized gut microbiota.

Exposure (E): Intervention with microbiota-modulating agents, specifically probiotics, prebiotics, psychobiotics, or targeted dietary interventions.

Comparison (C): Control groups with placebo administration, absence of intervention, or basal/normal condition.

Outcome (O): Modulation of the gut–brain axis communication, evaluated in different domains such as microbial, metabolic, immunological, neuroendocrine, and behavioral.

Owing to substantial methodological and clinical heterogeneity among the included studies, the findings were synthesized narratively; no meta-analysis was performed.

### 2.2. Focused Research Question

What are the effects of gut microbiota modulation through the administration of prebiotics, probiotics, and psychobiotics on the communication pathways of the gut microbiota–brain axis?

### 2.3. Search Strategy and Information Sources

A comprehensive, systematic literature search was conducted on 7 March 2026, in PubMed and Scopus to identify original studies investigating the modulation of the gut microbiota–brain axis by prebiotics, probiotics, and psychobiotics. As a supplementary source, Google Scholar was searched to identify potentially relevant studies, including gray literature, not retrieved through bibliographic databases. Screening was limited to the first 100 relevance-ranked results. This threshold was predefined as a stopping rule based on published guidance for search engines, which recommends restricting screening to a predetermined number of results, such as the first 100 [16].

The search strategies were developed using Medical Subject Headings (MeSH) terms for PubMed and equivalent keywords adapted to each database’s syntax. Boolean operators (AND and OR) were used to combine terms related to “Gastrointestinal Microbiome,” “Probiotics,” “Prebiotics,” and “Gut-Brain Axis.” The complete search strategies for each database are presented in Table 1.

### 2.4. Study Selection and Data Extraction

The records retrieved from the literature search were initially exported to the Zotero reference manager (version 7.0.32) for organization and subsequently imported into the Rayyan QCRI web platform (https://www.rayyan.ai, accessed on 11 March 2026), where duplicate records were identified and removed prior to screening.

Study selection was independently performed by two reviewers (S.R. and M.A.) in two consecutive phases, title and abstract screening and full-text assessment of potentially eligible studies, according to the predefined inclusion and exclusion criteria. Disagreements were resolved through discussion until consensus was reached.

Data extraction was independently conducted by the same reviewers (S.R. and M.A.) using a standardized data extraction form aligned with the review’s objectives. The extracted datasets were subsequently compared, and any discrepancies were resolved by consensus to ensure the accuracy and consistency of the collected information.

The extracted variables included the first author, publication year, study design, biological model, intervention characteristics (microbial strain or compound, dose, and duration), comparison group when applicable, and the main outcomes related to neurological function, behavioral responses, and the biological mechanisms underlying gut microbiota–brain axis communication.

### 2.5. Eligibility Criteria

#### 2.5.1. Inclusion Criteria

Original studies published between 2020 and 2026 involving humans or murine experimental models were included if they evaluated the effects of prebiotics, probiotics, or psychobiotics on gut microbiota–brain axis communication. Studies evaluating synbiotic formulations were also included because these combine prebiotic and probiotic components and are recognized as a complementary strategy for modulating the gut microbiota.

Eligible studies were required to evaluate at least one of the following outcomes, central or peripheral biomarkers, neurovagal signaling, endocrine parameters, immune responses, neurological function, or behavioral outcomes. Both preclinical studies and clinical trials were included to integrate the available mechanistic and translational evidence.

#### 2.5.2. Exclusion Criteria

Narrative reviews, systematic reviews, meta-analyses, study protocols, exclusively in vitro studies, studies conducted in non-mammalian models, and publications that did not provide sufficient information to characterize the evaluated interventions were excluded. Specifically, studies that did not identify the probiotic or psychobiotic strain(s) or adequately characterize the administered prebiotics were excluded.

### 2.6. Methodological Quality Assessment and Risk of Bias

To ensure a rigorous, study design-specific assessment, the included studies were appraised using three methodological quality and risk-of-bias instruments selected according to study design. The 48 in vivo animal studies were evaluated using the Systematic Review Center for Laboratory Animal Experimentation (SYRCLE) Risk of Bias tool [17]. The 27 randomized controlled clinical trials were assessed using the Joanna Briggs Institute (JBI) Critical Appraisal Checklist for Randomized Controlled Trials [18], The six non-randomized, pilot, or protocol-based clinical studies were evaluated using the JBI Critical Appraisal Checklist for Quasi-Experimental Studies [19].

Critical appraisal was performed independently by two reviewers (S.R. and M.A.), and any disagreements were resolved through discussion. Rather than relying on quantitative summary scores, the overall methodological quality and risk of bias were assessed using a qualitative, domain-based approach that considered the potential impact of unmet critical methodological domains on each study’s internal validity. The overall risk-of-bias judgment was reached by consensus among reviewers after collectively considering the relevance of all critical methodological domains, rather than by calculating summary scores or assigning equal weight to individual checklist items.

Studies were classified as having a low risk of bias when all critical domains were adequately addressed with no substantial threats to internal validity, a moderate risk of bias when one or two critical domains were unclear or unmet but the overall methodological rigor remained acceptable, and a high risk of bias when multiple unmet critical domains were considered likely to compromise the reliability of the reported findings. The results of the risk-of-bias assessment were visualized using the robvis web application [20].

### 2.7. Data Synthesis

Due to the substantial methodological heterogeneity among the included studies, arising from differences in biological models (human and rodent), intervention types (prebiotics, probiotics, psychobiotics, and synbiotic formulations), microbial strains, administered doses, intervention duration, and outcome measures, a meta-analysis was not feasible. Therefore, the findings were synthesized using a narrative approach.

The evidence was systematically organized in a structured data extraction matrix that compiled the following variables for each study, study identifier, study identification number, article title, authors, publication year, study design, biological model, intervention characteristics, treatment protocol (dose and duration), key outcomes, key observations, and the evaluated clinical condition.

The findings were subsequently integrated according to the biological mechanisms underlying gut microbiota–brain axis communication, considering the four principal signaling pathways described in the literature: neuronal, endocrine, immune, and metabolic. This approach enabled the identification of consistent patterns, areas of convergence and divergence, and emerging trends across the included studies while providing a comprehensive synthesis of their mechanisms of action and reported effects on neurological function and behavioral outcomes.

### 2.8. Assessment of Effect Measures, Reporting Bias, and Certainty of Evidence

Given the qualitative nature of this systematic review and the methodological heterogeneity of the included studies, quantitative pooled effect estimates were not calculated. Consequently, publication bias could not be formally assessed using graphical or statistical methods, such as funnel plots or Egger’s test, and the certainty of the evidence was not evaluated using the GRADE (Grading of Recommendations Assessment, Development and Evaluation) framework.

Instead, the strength of the available evidence was interpreted through the narrative synthesis and supported by study-specific methodological quality and risk-of-bias assessments conducted using the SYRCLE Risk of Bias tool and the Joanna Briggs Institute (JBI) Critical Appraisal tools, according to study design.

## 3. Results

### 3.1. Overview of the Included Studies

**Figure 1:**
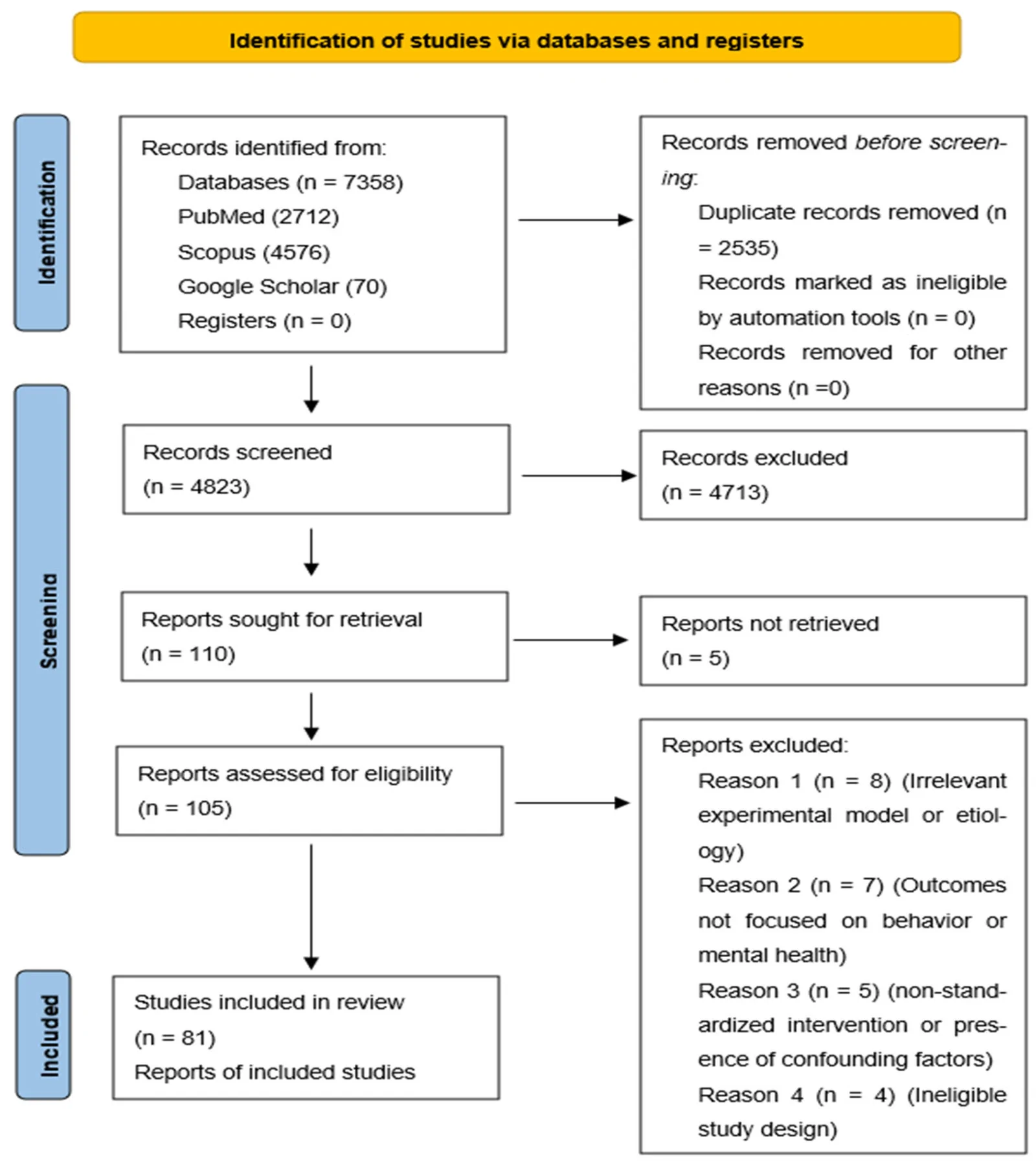
PRISMA 2020 flow diagram illustrating study identification, screening, eligibility assessment, and inclusion.

A systematic search of the PubMed, Scopus, and Google Scholar databases identified a total of 7358 records. After duplicate removal (n = 2535), 4823 records were screened based on their titles and abstracts. Subsequently, 110 reports were sought for retrieval, of which 5 could not be retrieved. A total of 105 full-text reports were assessed for eligibility, of which 24 were excluded for not meeting the predefined eligibility criteria. Ultimately, 81 original studies were included in the narrative synthesis, as illustrated in the PRISMA 2020 flow diagram (Figure 1).

The included studies were published between 2020 and 2026, with 72.8% published between 2022 and 2025. Based on their methodological design, the studies were classified into two main categories: 48 preclinical studies conducted in rodent experimental models [21,22,23,24,25,26,27,28,29,30,31,32,33,34,35,36,37,38,39,40,41,42,43,44,45,46,47,48,49,50,51,52,53,54,55,56,57,58,59,60,61,62,63,64,65,66,67,68], which primarily investigated the biological and molecular mechanisms underlying the gut microbiota–brain axis, and 33 human studies, including 27 randomized controlled trials and 6 non-randomized intervention studies [69,70,71,72,73,74,75,76,77,78,79,80,81,82,83,84,85,86,87,88,89,90,91,92,93,94,95,96,97,98,99,100,101], consisting predominantly of randomized, double-blind, placebo-controlled clinical trials, together with a smaller number of open-label intervention studies evaluating the clinical efficacy and translational potential of these interventions.

In the preclinical studies, human disorders—including depression, anxiety, autism spectrum disorder (ASD), epilepsy, and neurodegenerative diseases such as Alzheimer’s disease and Parkinson’s disease—were modeled using a variety of experimental approaches. These included pharmacologically induced models based on neurotoxins or other chemical agents [22,27,32,66], environmental models based on chronic unpredictable stress [30,61], and genetically engineered models that reproduced the neurobiological and behavioral characteristics of these disorders [21,24].

Considerable terminological and conceptual variability was observed across the included studies, with the terms psychobiotics, probiotic strains, and lactic acid bacteria often used interchangeably. Nevertheless, all interventions were selected according to a common functional criterion, namely, their demonstrated ability to modulate the gut microbiota–brain axis and produce measurable neurological or behavioral Outcome [45,46,64,100].

Among the interventions evaluated, probiotics were the predominant approach, including both single-strain and multi-strain formulations. Prebiotics represented the second most frequently investigated intervention, followed by synbiotic formulations, which combine live microorganisms with specific substrates to enhance gut microbiota modulation [46,56,69]. Detailed methodological and biological characteristics of the 81 included studies are presented in Table 2 and Table 3 and Supplementary Table S1.

### 3.2. Neurological Conditions Evaluated

The included studies were classified into four major categories of neurological and neuropsychiatric conditions (Table 4). The largest category comprised neurodegenerative disorders and cognitive enhancement (n = 31, 38.3%), including cognitive enhancement (n = 13, 16.0%) [34,44,50,65,67,79,84,85,87,91,94,98,99], Alzheimer’s disease (n = 10, 12.3%) [21,24,35,45,52,53,55,57,71,95], Parkinson’s disease (n = 7, 8.6%) [46,48,58,64,75,76,100], and Huntington’s disease (n = 1, 1.2%) [88].

The second category included mood disorders and stress-related disorders (n = 29; 35.8%), encompassing anxiety and depression (n = 23, 28.4%) [23,25,28,31,32,36,38,39,40,41,42,43,51,52,54,59,62,63,80,81,82,92,101], acute or chronic stress responses (n = 5, 6.2%) [30,62,77,89,90] and post-traumatic stress disorder (n = 1, 1.2%) [86].

The remaining studies addressed acute neurological, autoimmune, and behavioral conditions (n = 4, 4.9%), including seizures (n = 1, 1.2%) [56], epilepsy (n = 1, 1.2%) [37], multiple sclerosis (n = 1, 1.2%) [73] and substance use disorders (n = 1, 1.2%) [97]. Collectively, the included studies encompassed a broad spectrum of neurological and neuropsychiatric conditions in which gut microbiota modulation was evaluated.

The third category comprised neurodevelopmental disorders and other neuropsychiatric conditions (n = 17, 21.7%), predominantly autism spectrum disorder (ASD) (n = 13, 16.0%) [22,26,27,33,47,49,66,68,69,70,72,85,96], followed by attention-deficit/hyperactivity disorder (ADHD) (n = 2, 2.5%) [83,93], schizophrenia (n = 1, 1.2%) [29], and Tourette syndrome or tic disorders (n = 1, 1.2%) [74].

### 3.3. Intervention Strategies, Microbial Profiles, and Dosing Protocols

The included studies showed a clear predominance of microbiota-modulating interventions based on live microorganisms. Probiotics were the most frequently investigated approach (n = 61), including single-strain formulations (n = 35) [22,26,27,28,29,30,31,33,36,37,38,39,44,45,46,47,53,54,57,59,62,63,64,65,67,68,74,77,78,81,83,85,93,94,100] and multi-strain formulations (n = 26) [23,25,34,35,41,49,51,52,55,60,66,70,71,73,75,76,79,80,82,87,88,91,92,95,96,99]. Prebiotics represented the second most frequently investigated intervention (n = 12), [24,32,40,42,43,72,84,86,89,90,97,98] and were primarily administered as isolated functional substrates. Synbiotics (n = 8) [21,48,50,56,58,61,69,101] represented a complementary intervention strategy that combined live microorganisms with selectively utilized substrates to enhance modulation of the gut microbiota.

From a taxonomic perspective, the interventions were dominated by members of the family *Lactobacillaceae* (formerly represented under the genus *Lactobacillus*) and the genus *Bifidobacterium*, which were reported in 65 studies (n = 65, 80,2%) [21,22,23,25,26,27,28,29,30,33,34,35,36,37,38,39,41,44,45,46,47,48,49,50,51,52,53,54,55,56,58,59,60,61,63,64,65,66,67,68,69,70,71,73,74,75,76,77,78,79,80,81,82,83,85,87,88,91,93,94,95,96,99,100,101]. Several studies also evaluated emerging next-generation probiotic (NGP) candidates with potential to modulate the gut microbiota–brain axis, including *Akkermansia muciniphila* [31] and *Bacteroides uniformis* CECT 7771 [54]. Other investigations examined microorganisms with specific functional properties, including *Clostridium butyricum* [57], *Enterococcus faecalis EC-12* [62], and kefir-derived microbial consortia.

Most probiotic interventions administered doses ranging from 1 × 10^8^ to 1 × 10^10^ CFU/day, with 1 × 10^9^ CFU/day the most frequently reported dose across (n = 35, 43.2%) [25,26,27,28,29,30,31,33,34,35,36,37,38,39,44,45,46,47,48,49,50,51,53,54,56,57,58,59,61,63,64,65,66,67,68]. Prebiotic and synbiotic interventions predominantly employed fructooligosaccharides (FOSs), galactooligosaccharides (GOSs), and inulin as functional substrates [24,32,40,42,43,72,84,86,89,90,97,98]. Intervention duration ranged from short-term protocols (≤4 weeks) to long-term clinical and preclinical studies (>8 weeks), including prenatal and gestational interventions and prolonged dietary exposure protocols, depending on each study’s objectives and design.

### 3.4. Biological Mechanisms Underlying Gut Microbiota–Brain Axis Modulation

Although not all included studies explicitly identified the communication pathway through which the interventions exerted their effects, the reported biomarkers and outcome measures enabled the interventions to be classified into the four principal communication pathways of the gut microbiota–brain axis: neural, endocrine, immune, and metabolic. This classification was based on the primary biomarkers and outcome measures evaluated in each study, including pro-inflammatory cytokines for the immune pathway, cortisol or corticosterone for the endocrine pathway, and short-chain fatty acids (SCFAs) for the metabolic pathway.

The neural pathway was primarily associated with activation of the enteric nervous system and the vagus nerve. Clinical studies reported increased vagal tone following the administration of the interventions [24,74,82,92].

The endocrine pathway was characterized by modulation of the hypothalamic–pituitary–adrenal (HPA) axis, as evidenced by the normalization of salivary cortisol and serum corticosterone levels altered by chronic stress [25,36,57,77,78,79,80,90,99].

The immune pathway was among the most frequently reported mechanisms and was characterized by reduced levels of the pro-inflammatory cytokines TNF-α, IL-6, and IL-1β; attenuation of microglial activation; and preservation of gut and blood–brain barrier integrity through the regulation of key tight junction proteins, including claudins, occluding, and zonula occludens-1 (ZO-1) [23,26,27,30,35,39,40,45,48,50,51,58,59,61,65,70,71,75,76,85,93,94,95,96,101].

The metabolic pathway was primarily characterized by increased production of SCFAs (acetate, propionate, and butyrate) and tryptophan-derived metabolites, such as indole-3-propionic acid, which act as signaling molecules capable of stimulating the secretion of neuroprotective peptides, including glucagon-like peptide-1 [28,29,31,32,33,34,37,38,41,42,43,45,47,49,52,53,54,55,56,60,62,63,64,66,67,68,69,72,73,81,83,84,86,87,88,89,91,97,98,100].

### 3.5. Clinical Efficacy and Key Findings

Clinical studies reported significant improvements across a range of neurobehavioral outcomes, particularly adaptive behavior and social functioning, as assessed using the Autism Treatment Evaluation Checklist (ATEC) in individuals with autism spectrum disorder (ASD) [22,27,33,47,49,66,68]. However, reported efficacy requires balanced interpretation: neutral or conflicting outcomes were also observed regarding secondary metabolic and inflammatory markers, and statistical improvements did not consistently translate into clinically meaningful effects. In one small-scale trial, a psychobiotic intervention demonstrated exploratory improvements descriptively like clonidine with fewer adverse events [74]. Nonetheless, these findings require caution due to limited sample sizes and a lack of formal power calculations for clinical equivalence.

Additionally, preclinical studies have evaluated alternative delivery matrices, such as functional cookies, demonstrating high palatability and consistent daily intake in animal models [21]. However, these rodent studies monitored intake strictly through daily food consumption rather than objective clinical compliance metrics (e.g., pill counts or electronic monitoring).

### 3.6. Risk-of-Bias Assessment

The critical appraisal of the 81 included studies indicated an overall low-to-moderate risk of bias across all study designs, with no study classified as having a high risk of bias. Among the randomized controlled trials (RCTs; *n* = 27) assessed using the Joanna Briggs Institute (JBI) Critical Appraisal Checklist, 26 studies were judged to have a low risk of bias [70,72,74,76,77,78,80,81,82,84,85,86,87,88,89,90,91,92,94,95,96,97,98,99,100,101], whereas one pilot trial [75] was classified as having a moderate risk of bias due to limitations in allocation concealment and blinding.

All six quasi-experimental studies (*n* = 6) evaluated using the JBI Critical Appraisal Checklist for Quasi-Experimental Studies were classified as having a low risk of bias within the context of their non-randomized design [69,71,73,79,93,100]. Although these studies inherently lacked parallel control groups and participant blinding, they demonstrated clear cause-and-effect relationships, consistent pre- and post-intervention measurements, and adequate follow-up, supporting their overall methodological quality.

**Figure 2:**
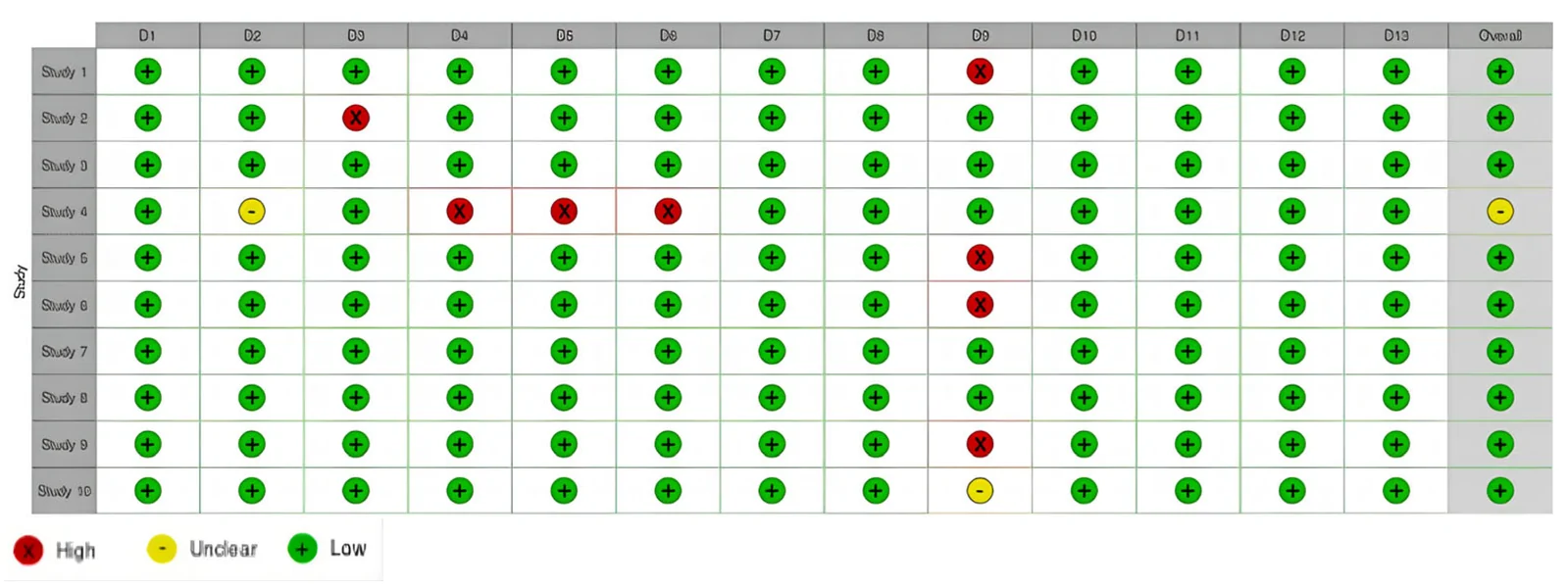
Summary of the critical appraisal and risk-of-bias assessment of human clinical studies (n = 27) evaluating gut microbiota–brain axis interventions.

**Figure 3:**
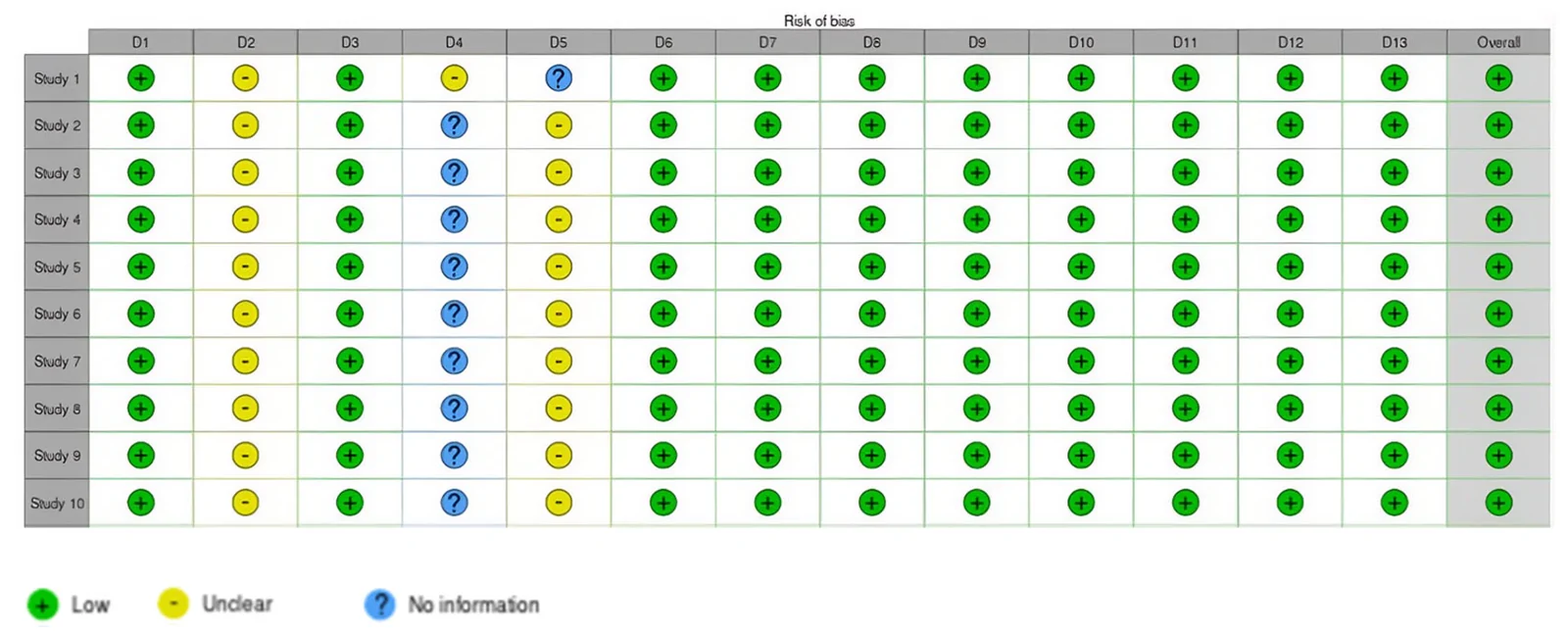
Summary of the critical appraisal and risk-of-bias assessment of preclinical studies in murine models (n = 48) evaluating gut microbiota–brain axis interventions.

**Figure 4:**
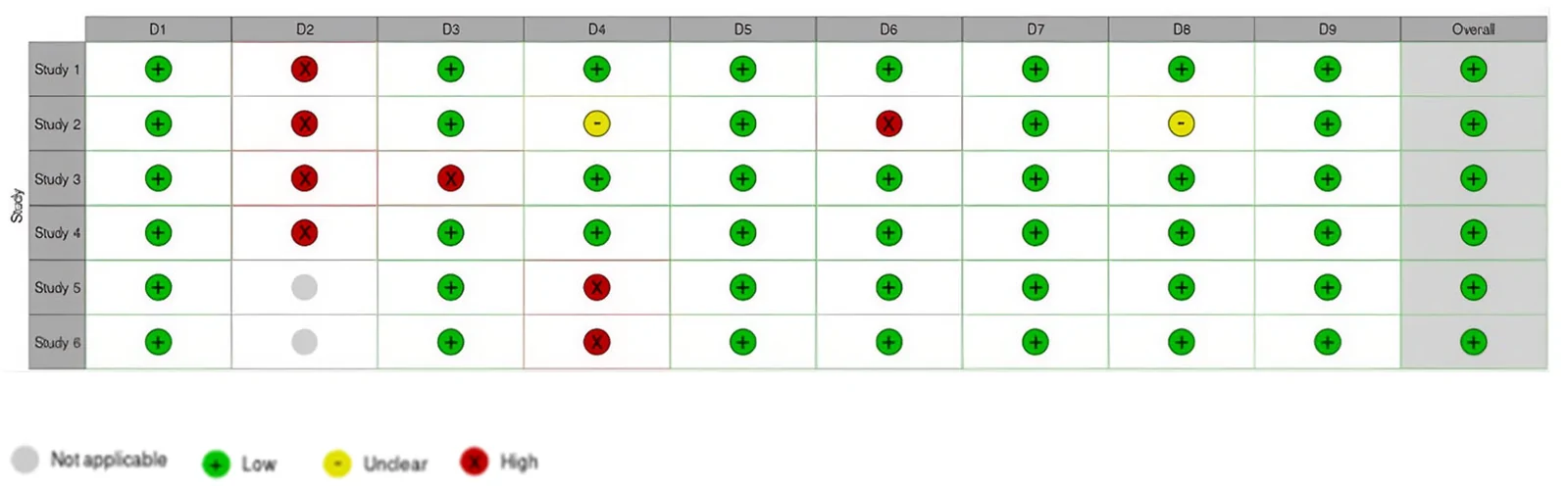
Summary of the critical appraisal and risk-of-bias assessment of clinical studies (n = 6) evaluating gut microbiota–brain axis interventions with quasi-experimental.

The 47 preclinical in vivo studies assessed using the SYRCLE Risk of Bias tool were likewise classified as having a low risk of bias [21,22,23,24,25,26,27,28,29,30,31,32,33,34,35,36,37,38,39,40,41,42,43,44,45,46,47,48,49,50,51,52,53,54,55,56,57,58,59,60,61,62,63,64,65,66,67,68]. The most frequently identified methodological limitations were sequence generation, allocation concealment, and blinding of caregivers or outcome assessors. Nevertheless, the studies consistently demonstrated comparable baseline characteristics, complete outcome reporting, and no evidence of selective outcome reporting, supporting their overall internal validity. Detailed domain-specific risk-of-bias assessments are presented in Figure 2, Figure 3 and Figure 4 and Supplementary Figure S1 and Figure S2.

Overall study-level risk of bias was synthesized based on the cumulative fulfillment of critical validity domains. Studies exhibiting unclear reporting in specific technical domains (e.g., sequence generation or blinding) were retained as low-to-moderate risk provided that key internal validity indicators—such as baseline comparability, complete outcome data, and absence of selective reporting—were fully satisfied. High risk of bias was reserved for studies demonstrating severe methodological flaws that directly compromised outcome interpretation.

## 4. Discussion

The primary objective of this systematic review was to critically synthesize the available evidence on the effects of probiotics, prebiotics, psychobiotics, and synbiotics on the gut microbiota–brain axis, with particular emphasis on the biological mechanisms underlying gut microbiota–brain communication and their implications for neurological and behavioral outcomes.

The findings reinforce the central role of the gut microbiota as an active regulator of gut microbiota–brain axis communication rather than a peripheral component of human physiology [1,14]. The high proportion of studies published between 2022 and 2025 (72.8%) reflects the rapid expansion of research in this field and the growing interest in microbiota-targeted interventions as complementary strategies to address some of the limitations of conventional psychopharmacology, particularly through the modulation of immune, metabolic, neuroendocrine, and behavioral processes involved in neurological and neuropsychiatric disorders [8,10,102].

Beyond confirming the therapeutic potential of microbiota modulation, this review also identified important research trends and persistent knowledge gaps. Most studies focused on neurodegenerative diseases and mood disorders, whereas considerably fewer investigated epilepsy, multiple sclerosis, Tourette syndrome, or substance use disorders. This imbalance highlights opportunities for future research while emphasizing the need to better understand the biological mechanisms underlying microbiota-mediated neuroregulation across a broader spectrum of neurological conditions [9,74].

One of the principal findings of this review is that psychobiotic activity should not be regarded as an intrinsic characteristic of an entire bacterial genus or species but rather as a strain-specific functional property. Although beneficial effects were reported across a wide range of neurological and neuropsychiatric disorders, the available evidence consistently indicates that neuroactive potential is determined by the unique genetic repertoire, metabolic capacity, and molecular mechanisms of individual strains rather than by taxonomic affiliation alone [103].

Accordingly, the therapeutic potential of psychobiotics should be established through functional and clinical validation of individual strains instead of being inferred solely from taxonomic classification [1,104]. This perspective also helps explain the considerable conceptual variability across the literature, where the terms psychobiotics, probiotic strains, and lactic acid bacteria are frequently used interchangeably despite representing biologically distinct concepts [1,11]. Collectively, these findings support a shift from empirical microorganism selection toward precision microbiome-based interventions guided by strain-specific functional evidence.

The importance of this strain-specific paradigm is illustrated by microorganisms in the family Lactobacillaceae (formerly classified under the genus *Lactobacillus*), which accounted for approximately 81.5% of the interventions included in this review yet exhibited markedly different biological activities. For example, *Lactiplantibacillus plantarum* N-1 selectively increased cecal butyrate and acetate production, resulting in improved sociability and reduced repetitive stereotypic behaviors in autism spectrum disorder models [45,46,68].

In contrast, *Lactiplantibacillus plantarum* DP189 primarily activated the PI3K/Akt/GSK-3β signaling pathway, reducing amyloid plaque accumulation while restoring hippocampal serotonin (5-HT) and γ-aminobutyric acid (GABA) concentrations in Alzheimer’s disease models [45]. These findings demonstrate that therapeutic efficacy depends on the functional properties of individual strains rather than on their taxonomic identity.

Collectively, these observations indicate that not all probiotics possess the functional capacity required to influence neurochemical signaling. Instead, psychobiotics should be considered a specialized functional subgroup whose therapeutic potential depends on experimentally demonstrated neurobiological activity. This conceptual framework supports the transition from empirical microorganism selection toward function-guided microbiota-based interventions and provides a more robust foundation for the development of precision microbiome therapeutics.

Although members of the family *Lactobacillaceae* (formerly represented under the genus *Lactobacillus*) and the genus *Bifidobacterium* remain the predominant microorganisms investigated because of their long-standing use as conventional probiotics, the evidence synthesized in this review indicates that microbiota-targeted interventions are becoming increasingly diverse. Emerging microbial candidates, including *Akkermansia muciniphila*, *Bacteroides uniformis*, and *Clostridium butyricum*, have demonstrated distinct biological mechanisms capable of modulating gut microbiota–brain axis communication, thereby expanding the repertoire of microorganisms with potential neuroactive properties [31,54,57].

Unlike conventional probiotics, whose benefits have traditionally been attributed to their broad effects on intestinal homeostasis, these next-generation microorganisms appear to exert more specialized functions. For example, *Akkermansia muciniphila* increased serotonin (5-HT) concentrations in both the prefrontal cortex and colon while simultaneously reducing systemic inflammation [31]. Likewise, *Clostridium butyricum* protected dopaminergic neurons through activation of the gut microbiota–GLP-1 signaling pathway in experimental models of Parkinson’s disease [57]. These observations illustrate the growing functional diversity of microbiota-based interventions and support continued exploration of novel microbial candidates for neurological disorders.

A similar principle applies to prebiotic and synbiotic interventions. Rather than acting directly on the central nervous system, prebiotics exert their effects indirectly by selectively stimulating resident microbial populations that produce neuroactive metabolites. Accordingly, fructooligosaccharides (FOSs) and galactooligosaccharides (GOSs) consistently promoted the expansion of bifidogenic communities, increased systemic acetate production, and attenuated hypothalamic–pituitary–adrenal (HPA) axis hyperactivity [21,24,32,40,42,43,48,50,56,58,61]. Likewise, synbiotic formulations combined the functional benefits of probiotics and selectively utilized substrates, potentially enhancing microbial survival, metabolic activity, and host responses beyond those achieved by either component alone.

Collectively, these findings indicate that successful modulation of the gut microbiota–brain axis depends on functional interactions within the microbial ecosystem rather than on the administration of individual microorganisms alone. This broader ecological perspective supports the development of microbiota-targeted interventions designed to optimize microbial functions and host responses simultaneously, providing a more comprehensive framework for future therapeutic strategies.

A major finding emerging from this review is that a single biological pathway cannot explain the therapeutic effects of microbiota-targeted interventions. Instead, the available evidence indicates that neuronal, endocrine, immune, and metabolic signaling pathways operate as an integrated communication network in which peripheral microbial signals ultimately converge to regulate neurological and behavioral function. Within this network, neuroinflammation appears to represent one of the principal biological nodes linking gut microbial activity with central nervous system responses [105].

When interpreting these biological pathways, preclinical animal models (n = 48) must be distinguished from human clinical evidence (n = 33). Animal studies serve strictly to establish biological plausibility and central neurochemical mechanisms, whereas clinical efficacy relies exclusively on human clinical trials. Within this network, neuroinflammation appears to represent one of the principal biological nodes linking gut microbial activity with central nervous system responses.

From a neuronal perspective, several studies have demonstrated that modulation of the gut microbiota directly influences gut–brain communication via the enteric nervous system and vagal signaling. Clinical studies using the multispecies formulation Ecologic^®^ BARRIER reported increased afferent vagal tone together with improvements in heart rate variability, an objective biomarker of gut microbiota–brain axis function [82]. Likewise, supplementation with inulin and fructooligosaccharides (FOS) promoted hippocampal neurogenesis, quantified by BrdU-positive cells, in chronic stress models [42].

The endocrine pathway also emerged as an important mediator of microbiota–brain communication. *Lacticaseibacillus paracasei* K56 reduced salivary cortisol concentrations and perceived stress in university students exposed to academic pressure [77]. Similarly, *Limosilactobacillus helveticus* (formerly *Lactobacillus helveticus*) NS8 normalized corticosterone and adrenocorticotropic hormone (ACTH) concentrations in a rat model of endogenous depression, supporting the capacity of microbiota-targeted interventions to modulate hypothalamic–pituitary–adrenal (HPA) axis activity [36].

In parallel, the immune pathway was the most consistently reported mechanism across the included studies. Microbiota-targeted interventions reduced circulating concentrations of TNF-α, IL-6, and IL-1β in autism spectrum disorder [22,70], Alzheimer’s disease [35,71], Parkinson’s disease [46,76], and depressive disorders [51,101]. Several studies also reported preservation of blood–brain barrier integrity through maintenance of the tight junction proteins claudin-5 and occludin, suggesting that attenuation of neuroinflammation constitutes a common mechanism linking microbial modulation with neurological improvement [40].

The metabolic pathway provided complementary evidence that the benefits of microbiota modulation extend beyond alterations in microbial composition and involve profound functional adaptations within the host. Increased production of short-chain fatty acids (SCFAs), particularly butyrate, acetate, and propionate, was consistently reported in models of lead-induced depression [52], autism spectrum disorder [22,68] and Alzheimer’s disease [53,60]. In addition, indole-3-propionic acid (IPA), a microbial metabolite derived from tryptophan metabolism, emerged as a promising neuroprotective biomarker in older adults with mild cognitive impairment [87].

Rather than operating independently, these pathways interact continuously. Microbial metabolites influence immune responses, inflammatory cytokines regulate neuroendocrine activity, and both ultimately affect neuronal communication through enteric and vagal signaling. This functional integration provides a plausible mechanistic explanation for the broad spectrum of neurological and behavioral improvements reported across the included studies. However, observed alterations in biological biomarkers—including inflammatory cytokines, salivary or circulating cortisol, short-chain fatty acids (SCFAs), and microbiota compositional shifts—must be interpreted as intermediate physiological correlates of exposure and biological activity rather than definitive proof of a direct causal mechanism.

In the absence of targeted mechanistic blockade or formal causal mediation analyses, these biological changes reinforce the concept of the gut microbiota–brain axis as a dynamic and multidirectional biological network operating through associative signaling pathways rather than established linear cause-and-effect mechanisms.

Collectively, these mechanisms support the gut microbiota’s role as a systemic regulator of neurodevelopment and neurodegeneration. They also provide a biological explanation for why several microbiota-targeted interventions achieved therapeutic effects comparable to those of selected first-line pharmacological treatments in specific preclinical and clinical settings while maintaining favorable safety profiles and fewer reported adverse effects [74].

Although the biological evidence supporting microbiota-targeted interventions continues to expand, their successful clinical translation remains limited by substantial methodological heterogeneity. Differences in microbial strains, intervention strategies, administered doses, treatment duration, delivery vehicles, and clinical endpoints complicate direct comparisons across studies and hinder the establishment of standardized therapeutic recommendations. This variability is particularly evident in dosing protocols, where 1 × 10^9^ CFU/day has become the most frequently administered dose within a broader range of 1 × 10^8^–1 × 10^10^ CFU/day, despite the absence of pharmacokinetic or pharmacodynamic justification in most studies [27,74].

The widespread empirical use of this dosing strategy may limit therapeutic optimization because probiotic efficacy depends on factors extending beyond the administered dose. Microbial survival during gastrointestinal transit varies according to the physiological characteristics of each strain and the formulation employed. Consequently, the same dose cannot be assumed to produce equivalent biological effects when delivered through different pharmaceutical formulations or food matrices. For example, 1 × 10^9^ CFU of *Lacticaseibacillus rhamnosus* administered as a capsule is unlikely to be functionally equivalent to the same dose incorporated into a food matrix that protects microbial viability during gastrointestinal transit [21].

Host-related factors also appear to influence therapeutic efficacy. Disease severity, baseline gut microbiota composition, and microbial interactions should all be considered when designing microbiota-based interventions. For instance, the combined administration of *Lacticaseibacillus rhamnosus* (formerly *Lactobacillus rhamnosus*) and Bifidobacterium longum produced greater anxiolytic and antidepressant effects than either strain administered individually, suggesting that clinical efficacy depends not only on dose but also on synergistic interactions between microorganisms [23]. These observations highlight the need for future dose–response studies incorporating host characteristics as key determinants of individualized therapeutic strategies.

The delivery vehicle itself also emerged as an important determinant of treatment success. A synbiotic red lentil cookie containing *Streptococcus thermophilus*, *Bifidobacterium lactis*, *Lactobacillus acidophilus*, *Limosilactobacillus helveticus* (formerly *Lactobacillus helveticus*), *Lacticaseibacillus paracasei*, *Lactiplantibacillus plantarum*, and *Levilactobacillus brevis* reduced amyloid-β deposition, improved spatial memory, and enhanced probiotic survival through the protective properties of the food matrix [21]. These findings demonstrate that formulation characteristics may directly influence therapeutic efficacy by improving microbial viability and functional activity.

This observation has clinical relevance for populations in which treatment adherence represents a major challenge, including individuals with autism spectrum disorder and other neurodevelopmental conditions. In these populations, the acceptability of the delivery vehicle may influence treatment success as strongly as the biological activity of the administered microorganisms.

Collectively, these findings indicate that successful clinical translation will require optimization of multiple interconnected factors rather than microbial strain selection alone. Future microbiota-based therapies should integrate strain-specific functionality, evidence-based dosing regimens, formulation design, delivery systems, and host characteristics into standardized therapeutic protocols. Such an approach will facilitate reproducibility, improve clinical efficacy, and accelerate the development of precision microbiome-based therapeutics [21,27].

One of the principal strengths of this systematic review is the integration of evidence from both preclinical and clinical studies, providing a comprehensive overview of how microbiota-targeted interventions influence communication along the gut microbiota–brain axis across a broad spectrum of neurological and neuropsychiatric disorders. By synthesizing findings from diverse experimental models and human studies, this review identified consistent biological patterns and highlighted mechanisms that appear conserved across pathological conditions.

Beyond summarizing the available evidence, this review provides an integrated conceptual framework for interpreting microbiota–brain communication. Rather than considering neuronal, endocrine, immune, and metabolic pathways as independent signaling routes, the synthesized evidence supports the view that these mechanisms function as an interconnected biological network in which neuroinflammation represents a major point of convergence. Furthermore, the findings consistently demonstrate that psychobiotic activity is a strain-specific functional property, reinforcing the importance of function-guided microorganism selection over taxonomic classification alone for future microbiota-based interventions.

Nevertheless, several limitations should be considered when interpreting these findings. Although the included studies generally exhibited high methodological quality and a low risk of bias, substantial heterogeneity in biological models, microbial strains, intervention strategies, dosing regimens, treatment duration, delivery systems, and outcome measures precluded quantitative synthesis through meta-analysis and limited direct comparisons across studies [15]. This heterogeneity is primarily driven by marked variations in intervention dosing, treatment duration, and the analytical resolution of microbial assessment methods. Consequently, identifying standardized therapeutic protocols and establishing evidence-based clinical recommendations remain challenging.

Another important limitation is the translational gap between preclinical and clinical research. Animal models induced by neurotoxins, chronic unpredictable stress, or genetic manipulation reproduce many mechanistic aspects of human neurological disorders. Still, they cannot fully capture their immunological, genetic, metabolic, and behavioral complexity. Accordingly, mechanistic findings derived from experimental models should be translated to clinical practice with appropriate caution until validated by well-designed randomized controlled trials [3,10].

Future research should prioritize harmonized methodological protocols, adequately powered randomized controlled trials, standardized outcome measures, strain-specific functional characterization, optimized dose–response studies, and improved delivery systems. Greater emphasis should also be placed on characterizing baseline gut microbiota composition and host-specific factors that may influence therapeutic responsiveness, thereby facilitating patient stratification and individualized intervention strategies.

Collectively, these advances will strengthen the reproducibility and clinical translation of microbiota-targeted therapies while supporting the transition toward precision microbiome-based therapeutics. Integrating microbial functionality, host biology, formulation science, and standardized clinical protocols will be essential to developing personalized interventions that maximize therapeutic efficacy across diverse neurological and neuropsychiatric disorders.

## 5. Conclusions

This systematic review indicates that communication along the gut microbiota–brain axis is best understood as a highly integrated biological network rather than a linear signaling process. The available evidence suggests that neuronal, endocrine, immune, and metabolic pathways interact dynamically, with neuroinflammation emerging as a major point of convergence. Modulation of pro-inflammatory cytokines, preservation of blood–brain barrier integrity, and production of neuroactive microbial metabolites appear to represent the principal biological mechanisms associated with favorable neurological outcomes.

The findings further indicate that psychobiotic activity is a strain-specific functional property rather than a general characteristic of probiotics. Consequently, future microbiota-based interventions should prioritize functional microbial characterization over taxonomic classification alone. Overall, while the available evidence suggests that psychobiotics, prebiotics, and synbiotics may be associated with favorable changes in behavioral and cognitive endpoints, their clinical efficacy and disease-specific applicability have not yet been clearly established.

Despite the overall acceptable methodological quality of the included studies, substantial heterogeneity in microbial strains, dosing strategies, intervention duration, formulations, and outcome measures continue to limit direct causal comparisons and the establishment of standardized clinical recommendations. Future research should prioritize harmonized methodological protocols, adequately powered randomized controlled trials, strain-specific functional characterization, optimized dose–response designs, standardized delivery systems, and clinically relevant outcome measures. Collectively, these findings support the transition from empirical microbiota-targeted interventions to precision microbiome-based therapeutic strategies that rely on strain-specific functional characterization, optimized formulations, and individualized host–microbiota interactions.

## Figures and Tables

**Table 1:** Search strategies used in PubMed, Scopus, and Google Scholar.

Database	Search Strategy
Scopus	TITLE-ABS-KEY ((“gut microbiota” OR “intestinal microbiome” OR “gut flora” OR “microbiome*”) AND (probiotic* OR prebiotic* OR psychobiotic* OR synbiotic*) AND (“gut-brain axis” OR “brain-gut axis” OR “gut-brain communication” OR neuroendocrin* OR behavior* OR behaviour*))
PubMed	((“Gastrointestinal Microbiome”[Mesh] OR “Microbiota”[Mesh] OR “Dysbiosis”[Mesh] OR “gut microbiota”[tiab] OR “intestinal microbiome”[tiab] OR “gut flora”[tiab] OR “enteric bacteria”[tiab])) AND (“Probiotics”[Mesh] OR “Prebiotics”[Mesh] OR “Synbiotics”[Mesh] OR “probiotic*”[tiab] OR “prebiotic*”[tiab] OR “psychobiotic*”[tiab] OR “synbiotic*”[tiab] OR “dietary fiber*”[tiab]) AND (“Gut-Brain Axis”[Mesh] OR “Brain-Gut Axis”[Mesh] OR “gut-brain axis”[tiab] OR “gut brain communication”[tiab] OR “microbiota-gut-brain interaction*”[tiab] OR “neuroendocrin*”[tiab] OR “behavior*”[tiab] OR “behaviour*”[tiab])
Google Scholar *	(“gut microbiota” OR “dysbiosis”) AND (“probiotics” OR “psychobiotics” OR “prebiotics”) AND (“gut-brain axis” OR “behavior”)

The asterisk (*) represents the wildcard truncation operator used in database search syntax to encompass word variations and plurals (e.g., microbiome*, probiotic*).

**Table 2:** Characteristics of the included studies.

N°	Title	Intervention	Intervention Detail Strain/Compound	Key Finding	Reference
1	A novel synbiotic (SCM06) for anxiety and sensory hyperresponsiveness in children with autism spectrum disorder: an open-label pilot study.	Synbiotic	SCM06 Mixture (*Bifidobacterium* and Prebiotics)	↓ Anxiety; ↓ Sensoru hyperreactivity; ↑ *B. pseudocatenulatum*	[69]
2	Kefir-derived probiotic mixture for children with autism spectrum disorder: a double-blind randomized clinical trial.	Probiotic (Multi-species)	Kefir-derived probiotic mixture (*Lactobacillus*, *Acetobacter*, *Kluyveromyces*).	↑ Vineland-3 (Adaptive behavior); ↓ TNF-α and IL-6	[70]
3	Microbiota–gut–brain axis dysregulation in Alzheimer’s disease and its modulation through probiotic supplementation.	Probiotic (Multi-species)	*B. lactis* W51, *B. lactis* W52, *L. acidophilus* W22, *L. paracasei* W20, *L. plantarum* W21, *L. salivarius* W24	↑ Cognitive scores (MMSE); ↓ Systemic inflammation markers	[71]

Note: ↑ indicates an increase (upregulation or improvement), whereas ↓ indicates a decrease (downregulation or reduction) in clinical outcomes, behavioral measures, or biological marker levels.

**Table 3:** Continued characteristics of the included studies.

N°	Title	Intervention	Intervention Detail Strain/Compound	Key Finding	Reference
4	A Double-Blind Randomised Controlled Trial of Prebiotic Supplementation in Children with Autism: Effects on Parental Quality of Life, Child Behaviour, Gastrointestinal Symptoms, and the Microbiome.	Prebiotic	Galacto-oligosaccharides (GOS)	Improvement in irritability and digestive symptoms; No significant changes in behavior; ↓ Mild GI symptoms	[72]
5	A probiotic approach identifies a Treg-centred immunoregulation via modulation of gut microbiota metabolites in people with multiple sclerosis and healthy individuals.	Probiotic (multi-species)	*Lactobacillus* * acidophilus*, *Lactobacillus* * plantarum*, *Lactobacillus par* * acasei*, *Lactobacillus del * *brueckii* subsp. *bulgaricus*, *Streptococcus thermophilus*, *Bifidobacterium breve*, *Bifido* * bacterium longum*, and * Bifidobacterium infantis*	↑ Frequency of peripheral treg cells and their suppressive capacity; ↑ Indole-3-acetate (IAA) levels; ↑ AhR metabolites	[73]
6	A red lentils-based synbiotic cookie exerts neuroprotective effects in a mouse model of Alzheimer’s disease.	Synbiotic	Red lentils and probiotic (*Streptococcus thermophilus*, *Bifidobacterium lactis*, *Lactobacillus acidophilus*, *Lactobacillus helveticus*, *Lactobacillus paracasei*, *Lactobacillus plantarum*, *Lactobacillus brevis*)	↓ Amyloid; ↑ Synaptic function; ↑ spatial memory; ↓ Aβ plaques and Tau; ↓ Neuroinflammation	[21]
7	Bifidobacterium adolescentis DM8504 Alleviates Autistic-Like Behaviors in Valproic Acid-Exposed Rats Through Gut Microbiota Modulation and SCFA Restoration.	Probiotic (Single strain)	*B. adolescentis* DM8504	↑ Sociability; ↑ Butyrate; ↓ IL-6 and TNF-α cytokines	[22]
8	Clinical Study of Limosilactobacillus reuteri for the Treatment of Children with Chronic Tic Disorders/Tourette Syndrome: A Mid-Term Efficacy Evaluation.	Probiotic (Single strain)	*Limosilactobacillus reuteri*	Efficacy like Clonidine; ↓ YGTSS score (Tics)	[74]

Note: ↑ indicates an increase (upregulation or improvement), whereas ↓ indicates a decrease (downregulation or reduction) in clinical outcomes, behavioral measures, or biological marker levels.

**Table 4:** Distribution of the included studies according to the main neurological conditions evaluated.

Neurological Condition Group	Condition Evaluated	Frequency (n)	Percentage (%)	References
Neurodegenerative diseases and cognitive optimization (n = 31) 38.3	Cognitive improvement	13	16.0%	[34,44,50,65,67,79,84,85,87,91,94,98,99]
	Alzheimer’s disease	10	12.3%	[21,24,35,45,52,53,55,57,71,95]
	Parkinson’s disease	7	8.6%	[46,48,58,64,75,76,100]
	Huntington’s disease	1	1.2%	[88]
Mood disorders and stress (n = 29) 35.8	Anxiety-depressive symptoms	23	28.4%	[23,25,28,31,32,36,38,39,40,41,42,43,51,52,54,59,62,63,80,81,82,92,101]
	Acute/chronic stress	5	6.2%	[30,61,77,89,90]
	Post-traumatic stress disorder	1	1.2%	[86]
Neurodevelopmental and neuropsychiatric spectrum disorders (n = 17) 21.0	Autism Spectrum Disorder	13	16.0%	[22,26,27,33,47,49,66,68,69,70,72,85,96]
	ADHD	2	2.5%	[83,93]
	Schizophrenia	1	1.2%	[29]
	Tourette syndrome/Tics	1	1.2%	[74]
Acute, autoimmune, and behavioral neurological conditions (n = 4) 4.9	Epilepsy	1	1.2%	[37]
	Seizures	1	1.2%	[56]
	Multiple Sclerosis	1	1.2%	[73]
	Addictions/substance use	1	1.3%	[97]

## Data Availability

The data analyzed in this study are fully synthesized and available within the body of the manuscript and its accompanying tables.

## Supplementary Materials

The following supporting information can be downloaded at https://www.mdpi.com/article/10.3390/biology15181598/s1, Supplementary Table S1: Characteristics of the included studies [21,22,23,24,25,26,27,28,29,30,31,32,33,34,35,36,37,38,39,40,41,42,43,44,45,46,47,48,49,50,51,52,53,54,55,56,57,58,59,60,61,62,63,64,65,66,67,68,69,70,71,72,73,74,75,76,77,78,79,80,81,82,83,84,85,86,87,88,89,90,91,92,93,94,95,96,97,98,99,100,101].; Supplementary Figure S1: Summary of the critical appraisal and risk-of-bias assessment of human clinical studies (n = 27) evaluating gut microbiota–brain axis interventions. Supplementary Figure S2: Summary of the critical appraisal and risk-of-bias assessment of preclinical studies in murine models (n = 48) evaluating gut microbiota–brain axis interventions. The following items are included in the supplementary materials: the PRISMA 2020 checklist for this study; the risk-of-bias matrices for preclinical and clinical studies (ROVis-Animal and ROVis-Human, respectively); the data extraction matrix for the studies (Systematic Review Microbiota); and, finally, Supplementary Table S1 in the document titled “Suplement”.
