# Supplementary material for: Modulation of the Gut Microbiota by Prebiotics, Probiotics, and Psychobiotics and Its Impact on the Gut Microbiota–Brain Axis: A Systematic Review
Source: Biology (Basel). 2026 Sep 10;15(18):1598. doi: 10.3390/biology15181598 (PMC13604514; doi:10.3390/biology15181598)
Supplement: Supplementary file 1 [file biology-15-01598-s001.zip › biology-4476321-supplementary.pdf]

biology

MDPI

Modulation of the Gut Microbiota by Prebiotics, Probiotics, and Psychobiotics and Its Impact on the Gut Microbiota–Brain Axis: A Systematic Review

Santiago Revelo [1,*] **and Miguel Anchundia** [1,2]

[1] Applied Microbiology Program, Universidad Politécnica Estatal del Carchi, Tulcán 040102, Carchi, Ecuador; harold.revelo@upec.edu.ec
[2] Food Engineering Program, Universidad Politécnica Estatal del Carchi, Tulcán 040102, Carchi, Ecuador; miguel.anchundia@upec.edu.ec
* Correspondence: harold.revelo@upec.edu.ec

Supplementary Table S1. Characteristics of the included studies.

N°	Title	Intervention	Intervention detail: Strain/Compound	Key Result	Reference
1	A novel Synbiotic (SCM06) for anxiety and sensory hyperresponsiveness in children with autism spectrum disorder: an open-label pilot study.	Synbiotic	Mixture SCM06 (*Bifidobacterium* + Prebiotics)	↓ Anxiety; ↓ Sensoru hyperreactivity; ↑ *B. pseudocatenulatum*	[19]
2	Kefir-derived probiotic mixture for children with autism spectrum disorder: a double-blind randomized clinical trial.	Probiotic (Multi-species)	Kefir-derived probiotic mixture (*Lactobacillus*, *Acetobacter*, *Kluyveromyces*)	↑ Vineland-3 (Adaptive behavior); ↓ TNF-α and IL-6	[20]
3	Microbiota-gut-brain axis dysregulation in Alzheimer's disease and its modulation through probiotic supplementation.	Probiotic (Multi-species)	*B. lactis* W51, *B. lactis* W52, *L. acidophilus* W22, *L. paracasei* W20, *L. plantarum* W21, *L. salivarius* W24	↑ Cognitive scores (MMSE); ↓ Systemic inflammation markers	[21]
4	A Double-Blind Randomized Controlled Trial of Prebiotic Supplementation in Children with Autism: Effects on Parental Quality of Life, Child Behaviour, Gastrointestinal Symptoms, and the Microbiome.	Prebiotic	Galactooligosaccharides (GOS)	Improvement in irritability and digestive symptoms; No significant changes in behavior; ↓ Mild GI symptoms	[22]
5	A probiotic approach identifies a Treg-centred	Probiotic (Multi-species)	*Lactobacillus acidophilus*, *Lactobacillus*	↑ Frequency of peripheral treg cells and their	[23]

	immunoregulation via modulation of gut microbiota metabolites in people with multiple sclerosis and healthy individuals		*plantarum, Lactobacillus par acasei, Lactobacillus del brueckii subsp. bulgaricus, Streptococcus thermophilus, Bifidobacterium breve, Bifido bacterium longum, and Bifidobacterium infantis*	suppressive capacity; ↑ Indole-3-acetate (IAA) levels; ↑ AhR metabolites	
**6**	A red lentils-based synbiotic cookie exerts neuroprotective effects in a mouse model of Alzheimer's disease.	Synbiotic	Red lentils + probiotic (*Streptococcus thermophilus*, *Bifidobacterium lactis*, *Lactobacillus acidophilus*, *Lactobacillus helveticus*, *Lactobacillus paracasei*, *Lactobacillus plantarum*, *Lactobacillus brevis*)	↓ Amyloid; ↑ Synaptic function; ↑ spatial memory; ↓ Aβ plaques and Tau; ↓ Neuroinflammation	[52]
**7**	Bifidobacterium adolescentis DM8504 Alleviates Autistic-Like Behaviors in Valproic Acid-Exposed Rats Through Gut Microbiota Modulation and SCFA Restoration.	Probiotic (Single strain)	*B. adolescentis* DM8504	↑ Sociability; ↑ Butyrate; ↓ IL-6 and TNF-α cytokines	[53]
**8**	Clinical Study of Limosilactobacillus reuteri for the Treatment of Children with Chronic Tic Disorders/Tourette Syndrome: A Mid-Term Efficacy Evaluation.	Probiotic (Single strain)	*Limosilactobacillus reuteri*	Efficacy like Clonidine; ↓ YGTSS score (Tics)	[24]
**9**	Combined Administration of Lactobacillus or Bifidobacterium Offers Enhanced Antidepressant and Anxiolytic Activity in a Dose Dependent Manner.	Probiotic (Multi-species)	*Lactobacillus acidophilus* y *Bifidobacterium longum*	↓ Immobility (TST/FST); greater effect with combined dose tan isolated	[54]
**10**	Dietary fiber modulates gut microbiota and lipid metabolism ameliorating Alzheimer's pathology in 5xFAD mice.	Prebiotic	Mixture: 1% Cellulose, 2% Pectin, 2% Inulin	↓ Aβ plaque load; ↓ Microglial activation; ↑ Neurogenesis	[55]
**11**	Effect of Probiotics Supplementation on REM Sleep Behavior Disorder and Motor Symptoms in Parkinson's Disease: A Pilot Study.	Probiotic (Multi-species)	*L. acidophilus, L. helveticus, L. rhamnosus, B. longum, B. infantis, B. lactis*	↓ REM sleep disorders; ↓ Motor Symptoms	[25]

**12**	Effects of a Four-Strain Probiotic on Gut Microbiota, Inflammation, and Symptoms in Parkinson's Disease: A Randomized Clinical Trial.	Probiotic (Multi-species)	Mixture of 4 strains (*L. acidophilus, L. paracasei, L. rhamnosus y B. animalis lactis*)	↓ Constipation; ↓ Systemic inflammatory markers; ↓ UPDRS-III score; ↓ Fecal calprotectin (inflammatory marker)	[26]
**13**	Effects of Lacticaseibacillus paracasei K56 on perceived stress among pregraduate students: a double-blind, randomized, placebo-controlled trial.	Probiotic (Single strain)	*Lacticaseibacillus paracasei* K56	↓ Perceived stress (PSS); ↓ Salivary cortisol; ↑ *Bifidobacterium* abundance	[27]
**14**	Effects of the Probiotics Lactobacillus rhamnosus DM163 and Lactobacillus delbrueckii DPUL-F36 on Anxiety-Related and Emotional Behaviors in Male Wistar Rats Under an Altered Light-Dark Cycle.	Probiotic (Multi-species)	*L. rhamnosus* DM163 o *L. delbrueckii* DPUL-F36	↓ Anxiety-like behavior; ↑ Exploration in the EPM; Mitigation of effects of circadian disruption	[56]
**15**	Efficacy and Safety of Lactobacillus delbrueckii subsp. lactis CKDB001 Supplementation on Cognitive Function in Mild Cognitive Impairment: A Randomized, Double-Blind, Placebo-Controlled Clinical Trial.	Probiotic (Single strain)	*Lactobacillus delbrueckii* subsp. *lactis* CKDB001	↑ Working memory; ↑ Sustained attention; ↑ Serum BDNF	[28]
**16**	ExoBDNF Probiotic Supplementation Enhances Cognition in Subjective Cognitive Decline.	Probiotic (Multi-species)	*L. plantarum*	↑ Working memory; ↑ Episodic memory; ↑ Serum BDNF levels	[29]
**17**	Exploring neurotransmitter regulation following probiotic supplementation in adults with subthreshold depression: A secondary analysis of a randomized controlled trial.	Probiotic (Multi-species)	*Limosilactobacillus fermentum* LF16 (DSM26956), *Lacticaseibacillus rhamnosus* LR06 (DSM21981), *Lactiplantibacillus plantarum* LP01 (LMG P-21021) and *Bifidobacterium longum* 04 (DSM23233)	↓ Depressive-like symptoms; ↑ Tryptophan; ↑ Plasma serotonin (5-HT) levels	[30]
**18**	Lacticaseibacillus rhamnosus GR-1 prevents autism-like behaviors by reshaping	Probiotic (Single strain)	*Lacticaseibacillus rhamnosus* GR-1	Prevention of tics and social deficits;	[57]

	the maternal and offspring microbiome.			Restored E/I balance in the mPFC	
**19**	Lacticaseibacillus rhamnosus SL42 ameliorates Wistar rat autistic-like behavior	Probiotic (Single strain)	*L. rhamnosus* SL42	↑ Sociability; ↓ Anxiety-like behavior; ↑ Grip strength	[58]
**20**	Lactiplantibacillus plantarum GOLDGUT-HNU082 Alleviates CUMS-Induced Depressive-like Behaviors in Mice by Modulating the Gut Microbiota and Neurotransmitter Levels.	Probiotic (Single strain)	Lactiplantibacillus plantarum GOLDGUT-HNU082	↑ 5-HT and BDNF; Restores microbial diversity	[59]
**21**	Lactiplantibacillus plantarum Lp815 decreases anxiety in people with mild to moderate anxiety: a direct-to-consumer, randomized, double-blind, placebo-controlled study	Probiotic (Single strain)	L. plantarum Lp815	↓ GAD-7 scores (Anxiety); ↑ Sleep quality	[31]
**22**	Lactobacillus Johnsonii YH1136 alleviates schizophrenia-like behavior in mice: a gut-microbiota-brain axis hypothesis study.	Probiotic (Single strain)	*Lactobacillus johnsonii* YH1136	↓ Psychomotor hyperactivity; ↑ Social interaction; ↑ Brain 5-HT levels	[60]
**23**	Limosilactobacillus fermentum 2L Ameliorates Chronic Stress-Induced Neuroinflammation through Gut-Brain Axis Modulation in Mice.	Probiotic (Single strain)	L. fermentum 2L	↓ Microglial activation; ↓ Pro-inflammatory cytokines	[61]
**24**	Multi-species probiotic supplement enhances vagal nerve function - results of a randomized controlled trial in patients with depression and healthy controls.	Probiotic (Multi-species)	Ecologic® BARRIER (*B. bifidum, B. lactis, L. acidophilus, L. brevis, L. casei, L. salivarius, L. lactis*)	↑ Heart rate variability (HRV); ↑ Vagal tone	[32]
**25**	Add-On Bifidobacterium Bifidum Supplement in Children with Attention-Deficit/Hyperactivity Disorder: A 12-Week Randomized Double-Blind Placebo-Controlled Clinical Trial.	Probiotic (Single strain)	*Bifidobacterium bifidum* BGN4	↓ Inattention and total ADHD-RS score; ↑ Quality of life	[33]

**26**	Akkermansia muciniphila Improves Depressive-Like Symptoms by Modulating the Level of 5-HT Neurotransmitters in the Gut and Brain of Mice	Probiotic (Single strain)	A. muciniphila (AKK)	↑ 5-HT levels in the prefrontal cortex and colon; ↓ Inflammation	[62]
**27**	Bacopaside, I alleviates depressive-like behaviors by modulating the gut microbiome and host metabolism in CUMS-induced mice.	Prebiotic	Bacopaside I	↑ SCFAs / Propionate; ↑ short-chain fatty acids, particularly acetic acid; ↑ *Firmicutes*, *Lactobacillus*, and *Streptococcus*	[63]
**28**	Effect of gut microbiome modulation on muscle function and cognition: the PROMOTe randomized controlled trial.	Prebiotic	Inulin y Fructooligosaccharides (FOS)	↑ Performance in memory and processing speed tests	[34]
**29**	Efficacy and safety of Bacteroides fragilis BF839 for pediatric autism spectrum disorder: a randomized clinical trial	Probiotic (Single strain)	*Bacteroides fragilis* BF839.	↓ CARS score (Autism severity); ↓ ABC score (Behavior); ↑ IL-10 levels (Anti-inflammatory)	
**30**	Enhancing social behavior in an autism spectrum disorder mouse model: investigating the underlying mechanisms of Lactiplantibacillus plantarum intervention.	Probiotic (Single strain)	*Lactiplantibacillus plantarum* PS128.	↑ Social interaction; ↑ Oxytocin in the hypothalamus	[64]
**31**	Prebiotics as an adjunct therapy for posttraumatic stress disorder: a pilot randomized controlled trial.	Prebiotic	Inulin, type 2 resistant starch, resistant maltodextrin, and rice bran	↓ PTSD symptoms (CAPS-5); ↑ *Akkermansia* abundance	[36]
**32**	Probiotic Lactobacilli Ameliorate Antibiotic-Induced Cognitive and Behavioral Impairments in Mice	Probiotic (Multi-species)	*L. acidophilus, L. casei, L. plantarum, L. rhamnosus*	↑ Recognition memory; ↓ Brain oxidative stress	[65]
**33**	Slowing Alzheimer's disease progression through probiotic supplementation.	Probiotic (Multi-species)	*Lactobacillus plantarum* KY1032 y *Lactobacillus curvatus* HY7601	↓ Hippocampal Aβ plaques; ↓ Microglial activation	[66]
**34**	Supplementation with Lactobacillus helveticus NS8 alleviated behavioral, neural, endocrine, and microbiota	Probiotic (Single strain)	*Lactobacillus helveticus* NS8	↓ Depressive-like behavior; ↑ 5-HT and NE; ↓ CORT and ACTH	[67]

	abnormalities in an endogenous rat model of depression.				
**35**	Gut microbiota indole-3-propionic acid mediates neuroprotective effect of probiotic consumption in healthy elderly: A randomized, double-blind, placebo-controlled, multicenter trial and in vitro study.	Probiotic (Multi-species)	*Bifidobacterium bifidum* BGN4 y *Bifidobacterium longum* BORI	↑ Cognitive scores; ↑ Indole-3-propionic acid (IPA)	[37]
**36**	A Randomized Controlled Trial of Probiotics Targeting Gut Dysbiosis in Huntington's Disease.	Probiotic (Multi-species)	*Lactobacillus rhamnosus, Saccharomyces cerevisiae, Bifidobacterium animalis ssp. Lactis*	Improved microbiota; no clinical change	[38]
**37**	Beneficial Effects of Probiotic Bifidobacterium longum in a Lithium-Pilocarpine Model of Temporal Lobe Epilepsy in Rats.	Probiotic (Single strain)	*Bifidobacterium longum*	↓ Seizure frequency; ↓ Neuronal death	[68]
**38**	Improvements of Age-Related Cognitive Decline in Mice by Lactobacillus helveticus WHH1889, a Novel Strain with Psychobiotic Properties.	Probiotic (Single strain)	*Lactobacillus helveticus* WHH1889.	↑ Spatial learning; ↑ Hippocampal 5-HT and GABA levels	[69]
**39**	Inulin-type fructans and 2'fucosyllactose alter both microbial composition and appear to alleviate stress-induced mood state in a working population compared to placebo (maltodextrin): the EFFICAD Trial, a randomized, controlled trial.	Prebiotic	Oligofructose (Inulin) and 2'-fucosyllactose (2'-FL)	↓ Perceived Stress Scale (PSS); ↑ *Bifidobacterium* abundance	[39]
**40**	Lactiplantibacillus plantarum APsulloc 331261 (GTB1TM) attenuates depressive-like behavior and physiology via association with gut microbiota in BALB/c mice	Probiotic (Single strain)	*L. plantarum Apsulloc* 331261 (GTB1)	↓ Helplessness (FST/TST); ↓ Hippocampal inflammation; ↑ Hippocampal 5-HT concentration	[70]

**41**	Lactiplantibacillus plantarum CR12 attenuates chronic unforeseeable mild stress induced anxiety and depression-like behaviors by modulating the gut microbiota-brain axis	Probiotic (Single strain)	*L. plantarum* CR12 y *L. plantarum* ST-1	↓ Neuroinflammation; ↓ Anxiety; ↓ Hippocampal pro-inflammatory cytokine expression	[71]
**42**	Lactiplantibacillus plantarum N-1 improves autism-like behavior and gut microbiota in mouse.	Probiotic (Single strain)	*Lactiplantibacillus plantarum* N-1	↑ Sociability; ↓ Repetitive behaviors; ↑ Butyrate and Acetate	[72]
**43**	Prebiotic Consumption Alters Microbiota but Not Biological Markers of Stress and Inflammation or Mental Health Symptoms in Healthy Adults: A Randomized, Controlled, Crossover Trial.	Prebiotic	Fructooligosaccharides (FOS) and Galactooligosaccharides (GOS)	No changes in cortisol or inflammation; ↑ *Bifidobacterium*	[40]
**44**	Probiotic intervention benefits multiple neural behaviors in older adults with mild cognitive impairment.	Probiotic (Multi-species)	*Lactobacillus plantarum, Lactococcus lactis, Bifidobacterium lactis, Lactobacillus johnsonii, Lactobacillus paracasei, Lactobacillus salivarius, Lactobacillus acidophilus, Lactobacillus rhamnosus, Bifidobacterium animalis, Lactobacillus casei, Lactobacillus reuteri, Lactobacillus fermentum, Bifidobacterium infantis*	↑ Global cognitive function; ↓ Anxiety; ↑ MoCA and RBANS scores; ↑ *Bacteroides* abundance; ↑ BDNF concentration	[41]
**45**	Sex difference in prebiotics on gut and blood-brain barrier dysfunction underlying stress-induced anxiety and depression.	Prebiotic	Fructooligosaccharides (FOS).	↓ Anxiety (more pronounced in females); ↑ Tight junction proteins (ZO-1, Occludin)	[73]
**46**	Suppressive Effects of Lactobacillus on Depression through Regulating the Gut Microbiota and Metabolites in C57BL/6J Mice Induced by Ampicillin.	Probiotic (Multi-species)	(*L. rhamnosus* GMNL-74, *L. acidophilus* GMNL-185, and *L. plantarum* GMNL-141	↓ Depressive-like behavior; ↑ BDNF expression; ↑ Butyrate	[74]
**47**	The Influence of Topinambur and Inulin Preventive Supplementation on Microbiota, Anxious Behavior,	Prebiotic	Inulin y Topinambur	↑ Hippocampal neurogenesis (BrdU+ cells); ↓ Anxiety	[75]

	Cognitive Functions and Neurogenesis in Mice Exposed to the Chronic Unpredictable Mild Stress.				
**48**	Administration of xylo-oligosaccharides improves depressive-like behavior in mice caused by chronic unpredictable mild stress by altering microbiota composition	Prebiotic	Xylo-oligosaccharides (XOS)	↑ Sucrose preference; ↑ Brain 5-HT and GABA levels	[76]
**49**	Clinical, gut microbial and neural effects of a probiotic add-on therapy in depressed patients: a randomized controlled trial.	Probiotic (Multi-species)	*Streptococcus thermophilus, Bifidobacterium breve, Bifidobacterium longum, Bifidobacterium infantis, Lactobacillus acidophilus, Lactobacillus plantarum, Lactobacillus paracasei,* *L. helveticus*	↓ HAM-D score; ↑ Insula activation to emotional stimuli	[42]
**50**	Effect of Bifidobacterium bifidum on Clinical Characteristics and Gut Microbiota in Attention-Deficit/Hyperactivity Disorder.	Probiotic (Single strain)	*Bifidobacterium bifidum* BGN4	↓ Inattention and Anxiety; ↑ Microbial diversity (*Firmicutes*)	[43]
**51**	Effects of Lactiplantibacillus plantarum OLL2712 on Memory Function in Older Adults with Declining Memory: A Randomized Placebo-Controlled Trial.	Probiotic (Single strain)	*L. plantarum OLL2712 (heat-treated cells)*	↑ Visual memory and RBANS score; ↓ Systemic inflammation	[44]
**52**	Effects of probiotic supplements on cognition, anxiety, and physical activity in subjects with mild and moderate Alzheimer's disease: A randomized, double-blind, and placebo-controlled study.	Probiotic (Multi-species)	*Lactobacillus* y *Bifidobacterium* (*L. acidophilus*, *L. casei*, *L. fermentum* y *B. bifidum*).	↑ MMSE (Cognition); ↓ Anxiety; ↓ Inflammatory markers	
**53**	Lactobacillus paracasei PS23 improves cognitive deficits via modulating the hippocampal gene expression and the gut microbiota	Probiotic (Single strain)	*L. paracasei* PS23 *y* HK-PS23	↓ Cognitive impairment; ↓ Inflammation	[77]

	in D-galactose-induced aging mice.				
**54**	Lactobacillus plantarum DP189 prevents cognitive dysfunction in D-galactose/AlCl (3) induced mouse model of Alzheimer's disease via modulating gut microbiota and PI3K/Akt/GSK-3β signaling pathway.	Probiotic (Single strain)	*L. plantarum* DP189	↑ Spatial memory; ↓ Aβ plaques; ↑ 5-HT and GABA levels; ↑ SOD/GSH-Px	[78]
**55**	Lactobacillus plantarum DP189 Reduces α-SYN Aggravation in MPTP-Induced Parkinson's Disease Mice via Regulating Oxidative Damage, Inflammation, and Gut Microbiota Disorder.	Probiotic (Single strain)	*L. plantarum* DP189	↓ α-synuclein aggregates; ↑ Dopamine; weeks; ↓ Pro-inflammatory cytokines (IL-6, TNF-α)	[79]
**56**	Lactobacillus plantarum ST-III modulates abnormal behavior and gut microbiota in a mouse model of autism spectrum disorder.	Probiotic (Single strain)	*L. plantarum ST-III*	↑ Sociability; ↓ Anxiety; ↑ Microbiota diversity	[80]
**57**	Polymannuronic acid prebiotic plus Lacticaseibacillus rhamnosus GG probiotic as a novel synbiotic promoted their separate neuroprotection against Parkinson's disease.	Synbiotic	Polymannuronic acid (PM) + *L. rhamnosus* GG (LGG)	↑ TH+ neurons in SNpc; ↓ α-synuclein; ↑ Butyrate	[81]
**58**	Randomized Double-Blind Crossover Study for Evaluating a Probiotic Mixture on Gastrointestinal and Behavioral Symptoms of Autistic Children	Probiotic (Multi-species)	*Bifidobacterium breve* BR03; *Bifidobacterium junctum* LP01; *Lactobacillus plantarum* LP01.	↓ Irritability and lethargy (ABC scale); ↓ GI symptoms	[46]
**59**	Restoring an adequate dietary fiber intake by inulin supplementation: a pilot study showing an impact on gut microbiota and sociability in alcohol use disorder patients.	Prebiotic	Inulin	↑ Sociability; ↓ Craving; ↑ *Bifidobacterium*	[47]

**60**	Selective Probiotic Treatment Positively Modulates the Microbiota-Gut-Brain Axis in the BTBR Mouse Model of Autism.	Probiotic (Multi-species)	*Lactobacillus plantarum* LP01.	↑ Social behavior; ↓ Anxiety; ↑ Tryptophan metabolites	[82]
**61**	Selenium Nanoparticles-Enriched Lactobacillus casei ATCC 393 Prevents Cognitive Dysfunction in Mice Through Modulating Microbiota-Gut-Brain Axis.	Synbiotic	*Lactobacillus casei* ATCC 393	Improved spatial memory; ↓ Aβ and Phospho-Tau plaques; ↓ Mitochondrial damage	[83]
**62**	The Alleviation of Gut Microbiota-Induced Depression and Colitis in Mice by Anti-Inflammatory Probiotics NK151, NK173, and NK175.	Probiotic (Multi-species)	*Bifidobacterium longum* NK151, *Lactobacillus mucosae* NK173 y *Bifidobacterium bifidum* NK175.	Depressive-like behavior; ↓ NF-κB expression; ↓ TNF-*α*	[84]
**63**	The role of probiotic intervention in regulating gut microbiota, short-chain fatty acids and depression-like behavior in lead-exposed rats.	Probiotic (Multi-species)	*Lactobacillus* y *Bifidobacterium*	Depressive-like behavior; ↑ Fecal acetate and butyrate	[85]
**64**	A specific dietary fibre supplementation improves cognitive performance-an exploratory randomized, placebo-controlled, crossover study.	Prebiotic	Polydextrose (PDX)	↑ Working memory; ↓ Reaction time; ↑ *Ruminococcus*	[48]
**65**	Administration of Bifidobacterium breve Improves the Brain Function of Aβ (1-42)-Treated Mice via the Modulation of the Gut Microbiome.	Probiotic (Single strain)	*Bifidobacterium breve* (CCFM1025)	↑ Y-maze performance; ↑ Fecal butyrate; ↓ Synaptic damage	[86]
**66**	Bacteroides uniformis CECT 7771 Modulates the Brain Reward Response to Reduce Binge Eating and Anxiety-Like Behavior in Rat	Probiotic (Single strain)	*B. uniformis* CECT 7771	↓ Anxiety; ↓ Binge eating	[87]

**67**	Impact of interval training with probiotic (L. plantarum / Bifidobacterium bifidum) on passive avoidance test, ChAT and BDNF in the hippocampus of rats with Alzheimer's disease	Probiotic (Multi-species)	*L. plantarum* + *B. bifidum*	↑ Memory; ↑ Hippocampal BDNF and ChAT	[88]
**68**	Neuroprotective effect of both synbiotics and ketogenic diet in a pentylenetetrazol-induced acute seizure murine model.	Synbiotic	*L. fermentum* MSK 408 + GOS	↓ Seizure frequency; ↑ GABA/Glutamate ratio	[89]
**69**	Probiotic Clostridium butyricum ameliorated motor deficits in a mouse model of Parkinson's disease via gut microbiota-GLP-1 pathway.	Probiotic (Single strain)	*Clostridium butyricum*	↓ Motor deficit; Protects DA neurons; ↑ Serum and brain GLP-1 levels; ↑ BDNF	[90]
**70**	Probiotic Supplementation Improves Cognitive Function and Mood with Changes in Gut Microbiota in Community-Dwelling Older Adults: A Randomized, Double-Blind, Placebo-Controlled, Multicenter Trial.	Probiotic (Multi-species)	*Bifidobacterium bifidum* BGN4 Bifidobacterium *longum Bifidobacterium bifidum* BGN4 and *Bifidobacterium longum* BORI	↑ Memory and attention; ↓ Stress; ↓ Blood stress markers	[49]
**71**	The Add-On Effect of Lactobacillus plantarum PS128 in Patients With Parkinson's Disease: A Pilot Study	Probiotic (Single strain)	*L. plantarum* PS128	↓ UPDRS score (Motor); ↑ Quality of life (PDQ-39)	[50]
**72**	The impact of dextran sodium sulphate and probiotic pre-treatment in a murine model of Parkinson's disease.	Synbiotic	*Bifidobacterium, Lactobacillus, Streptococcus*	↓ TH+ neuron loss (only partial); ↓ Intestinal inflammatory markers	[91]
**73**	A Probiotic Lactobacillus gasseri Alleviates Escherichia coli-Induced Cognitive Impairment and Depression in Mice by Regulating IL-1β Expression and Gut Microbiota.	Probiotic (Single strain)	*Lactobacillus gasseri* NK109.	↑ Memory; ↓ Brain IL-1β expression; ↑ BDNF	[92]

**74**	Effects of Probiotic Supplementation on Short Chain Fatty Acids in the AppNL-G-F Mouse Model of Alzheimer's Disease.	Probiotic (Multi-species)	VSL#3 (*B. breve, B. longum, B. infantis, L. acidophilus, L. plantarum, L. paracasei, L. bulgaricus, S. thermophilus*)	↑ Fecal butyrate and acetate; ↓ Cortical microglial activation	[93]
**75**	Glycated milk protein fermented with Lactobacillus rhamnosus ameliorates the cognitive health of mice under mild-stress condition	Synbiotic	*L. rhamnosus + glycated milk protein*	↑ Hippocampal BDNF expression; ↓ Anxiety; ↑ Memory	[94]
**76**	Heat-killed Enterococcus fecalis (EC-12) supplement alters the expression of neurotransmitter receptor genes in the prefrontal cortex and alleviates anxiety-like behavior in mice.	Probiotic (Single strain)	*E. faecalis* EC-12 *(Inactive)*	↓ Anxiety; Modulates PFC receptors	[95]
**77**	Lactobacillus casei improves depression-like behavior in chronic unpredictable mild stress-induced rats by the BDNF-TrkB signal pathway and the intestinal microbiota.	Probiotic (Single strain)	*L. casei*	↓ Depressive-like behavior; Restored microbiota structure	[96]
**78**	Lactobacillus plantarum PS128 alleviates neurodegenerative progression in 1-methyl-4-phenyl-1,2,3,6-tetrahydropyridine-induced mouse models of Parkinson’s disease.	Probiotic (Single strain)	*L. plantarum* PS128	↓ Dopaminergic neuron loss; ↓ Oxidative stress	[97]
**79**	Probiotic Lactobacillus fermentum strain JDFM216 improves cognitive behavior and modulates immune response with gut microbiota.	Probiotic (Single strain)	*Lactobacillus fermentum* JDFM216	↑ Spatial memory; ↑ Macrophage activity; ↓ Anhedonia	[98]
**80**	PROVIT: Supplementary Probiotic Treatment and Vitamin B7 in Depression-A Randomized Controlled Trial.	Synbiotic	*B. bifidum, B. lactis, B. lactis, L. acidophilus, L. casei, L. paracasei, L. plantarum, L. salivarius, and L. lactis; 125 mg of D-Biotin (vitamin B7), 30 mg of common horsetail, 30 mg of fish collagen, and 30 mg of keratin matrix.*	↓ Depressive symptoms; ↓ Inflammatory markers (IL-6)	[51]

**81**	Supplementation of lactobacillus probiotic strains supports gut-brain-axis and defends autistic deficits occurred by valproic acid-induced prenatal model of autism	Probiotic (Multi-species)	*L. Plantarum- UBLP-40 (MTCC 5380), L. Casei- UBLC-42 (MTCC 5381), L. Acidophilus- UBLA-34 (MTCC 5401), L. Bulgaricus- Lactobacillus bulgaricus UBLB-38;*	↑ Sociability; ↓ Brain oxidative stress; ↑ Acetylcholinesterase activity	[99]

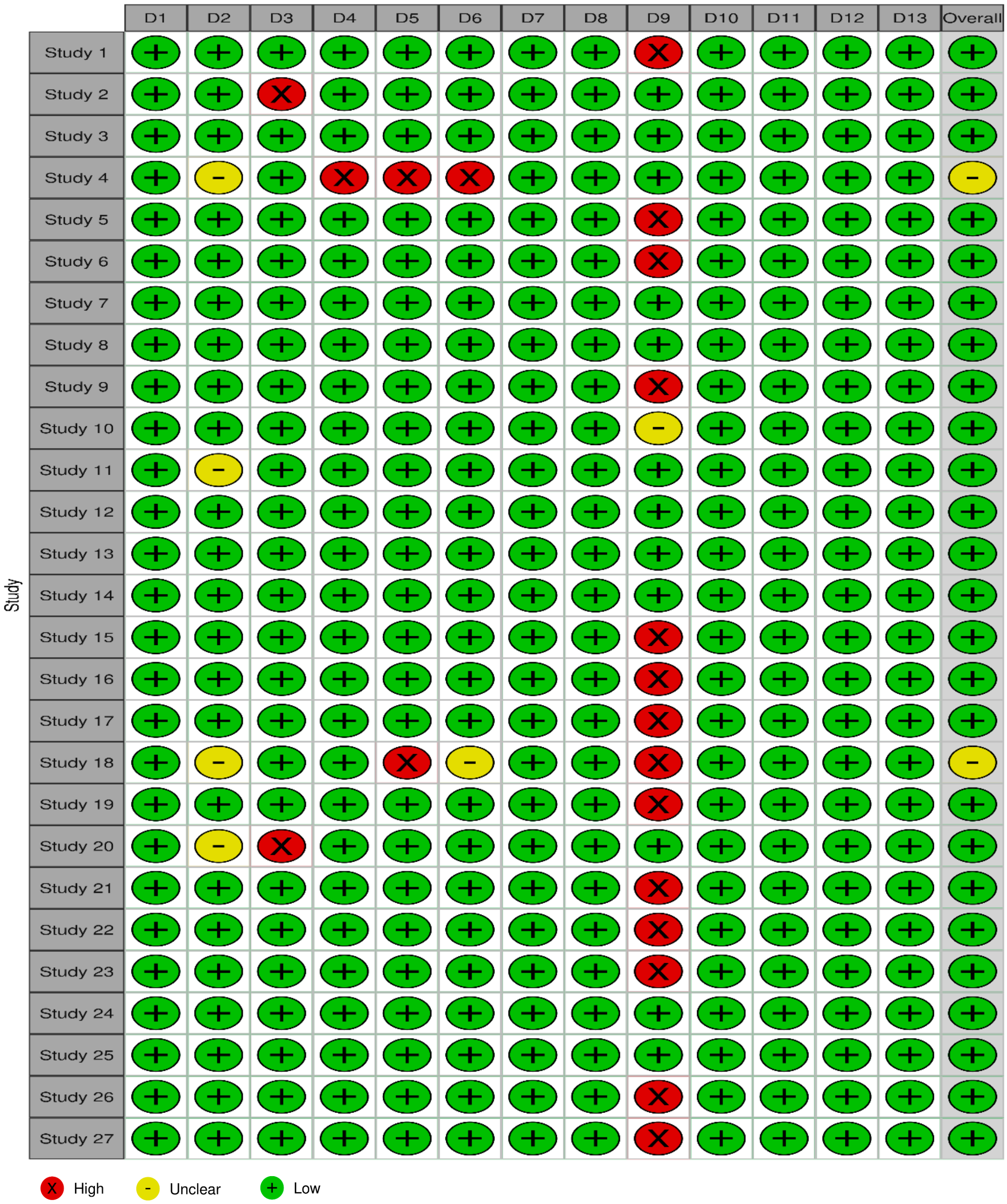

Supplementary Figure S1. Item-by-item methodological quality assessment of the included human studies using the Joanna Briggs Institute (JBI) critical appraisal tool.

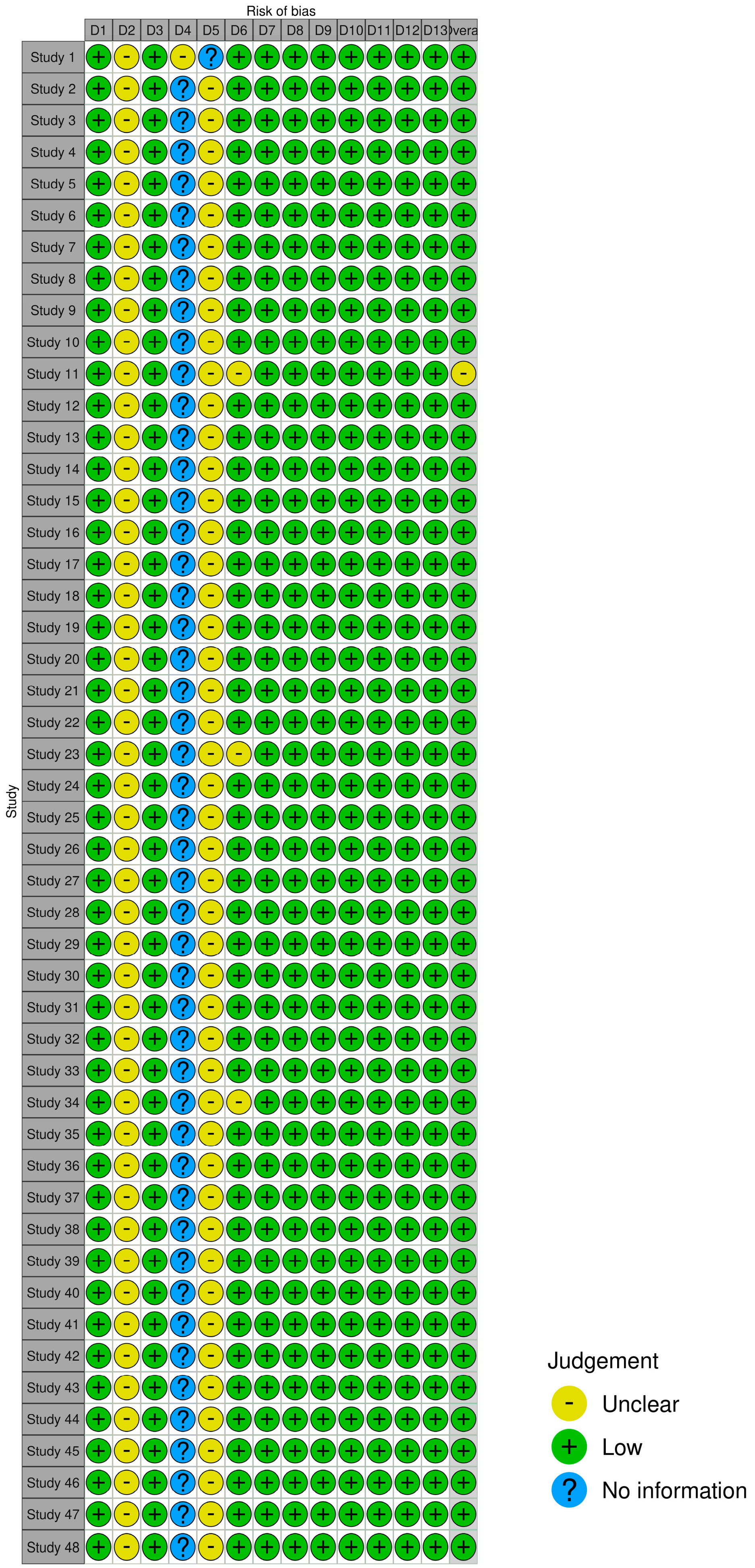
Risk of bias
D1
D2
D3
D4
D5
D6
D7
D8
D9
D10
D11
D12
D13
Overall
Study
Study 1
Study 2
Study 3
Study 4
Study 5
Study 6
Study 7
Study 8
Study 9
Study 10
Study 11
Study 12
Study 13
Study 14
Study 15
Study 16
Study 17
Study 18
Study 19
Study 20
Study 21
Study 22
Study 23
Study 24
Study 25
Study 26
Study 27
Study 28
Study 29
Study 30
Study 31
Study 32
Study 33
Study 34
Study 35
Study 36
Study 37
Study 38
Study 39
Study 40
Study 41
Study 42
Study 43
Study 44
Study 45
Study 46
Study 47
Study 48
Judgement
Unclear
Low
No information

Supplementary Figure S2. Summary of the critical appraisal and risk-of-bias assessment of preclinical studies in murine models (n = 49) evaluating gut microbiota–brain axis interventions.
